# Unlocking the specificity of antimicrobial peptide interactions for membrane-targeted therapies

**DOI:** 10.1016/j.csbj.2024.04.022

**Published:** 2024-04-12

**Authors:** Daniel Conde-Torres, Martín Calvelo, Carme Rovira, Ángel Piñeiro, Rebeca Garcia-Fandino

**Affiliations:** a Center for Research in Biological Chemistry and Molecular Materials, Departamento de Química Orgánica, Universidade de Santiago de Compostela, Campus Vida s/n, 15782 Santiago de Compostela, Spain; b Departamento de Física Aplicada, Facultade de Física, Universidade de Santiago de Compostela, 15782 Santiago de Compostela, Spain; c Departament de Química Orgànica and Institut de Química Teòrica i Computacional (IQTCUB), Universitat de Barcelona, Barcelona, Spain; d Institució Catalana de Recerca i Estudis Avançats (ICREA), Barcelona, Spain

**Keywords:** Antimicrobial peptides, Molecular dynamics, Lipid membranes, Enhanced sampling methods, Metadynamics, Potential of Mean Force (PMF), Free energy surface (FES)

## Abstract

Antimicrobial peptides (AMPs) are increasingly recognized as potent therapeutic agents, with their selective affinity for pathological membranes, low toxicity profile, and minimal resistance development making them particularly attractive in the pharmaceutical landscape. This study offers a comprehensive analysis of the interaction between specific AMPs, including magainin-2, pleurocidin, CM15, LL37, and clavanin, with lipid bilayer models of very different compositions that have been ordinarily used as biological membrane models of healthy mammal, cancerous, and bacterial cells. Employing unbiased molecular dynamics simulations and metadynamics techniques, we have deciphered the intricate mechanisms by which these peptides recognize pathogenic and pathologic lipid patterns and integrate into lipid assemblies. Our findings reveal that the transverse component of the peptide's hydrophobic dipole moment is critical for membrane interaction, decisively influencing the molecule's orientation and expected therapeutic efficacy. Our approach also provides insight on the kinetic and dynamic dependence on the peptide orientation in the axial and azimuthal angles when coming close to the membrane. The aim is to establish a robust framework for the rational design of peptide-based, membrane-targeted therapies, as well as effective quantitative descriptors that can facilitate the automated design of novel AMPs for these therapies using machine learning methods.

## Highlights

• Novel insights into antimicrobial peptides (AMPs) and lipid membranes interaction.
• Unveiling AMPs' role in combating infections and targeting cancer cells.
• Use of unbiased molecular dynamics and metadynamics in AMP research.
• Revealing key roles of hydrophobic dipole moments in antimicrobial action.
• Establishing a framework for rational peptide therapy design and development.

## Graphical Abstract

**Figure:**
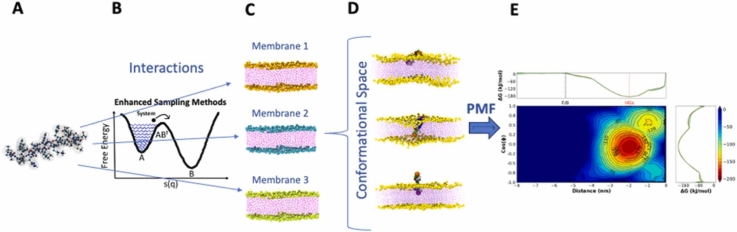
**Caption:** Schematic graphic representation of the work performed. **A)** AMP (magainin-2) and **C)** membrane models used in the study. **B)** Example of a sampling optimization algorithm based on metadynamics (metaD) that allows exploration of a space of conformations defined by the selected collective variables. **D)** Different snapshots observed in a metadynamics simulation that would not be observed in an unbiased MD simulation. **E)** 2D-Graphical representation of the interaction free energy surface (PMF) obtained in one of the systems used.

## 1 Introduction

Lipid membranes, with a thickness of just about four nanometers, delineate the boundary between life and death in biological cells [1]. These structures represent intricate and dynamic systems comprised of lipids in specific ratios influenced by various factors including diet, age, and environmental exposure [2]. A growing body of research reveals that many pathological cells contain substantial alterations in their lipid membrane composition compared to their healthy counterparts. Such changes are evident in cells associated with cancer [3], inflammation [4], bacterial and viral infections [5], and cellular aging [6]. Moreover, continual exposure to infections by select viruses, bacteria, and fungi [7] has been linked to inflammation, further augmenting the risk of cancer development [8]. This relationship positions lipidomics as a potential convergent point between these conditions, which are traditionally treated as distinct entities [9], [10]. The emerging perspective that lipid membrane composition is a viable target for therapy is poised to innovate treatment paradigms. Focusing on the lipidic component of cells provides a strategic alternative to the conventional protein receptor-based approaches and presents a novel pathway to counteract the escalating problem of drug resistance across a myriad of pathogens and pathologic conditions.

In the realm of lipidomics, Antimicrobial Peptides (AMPs) stand out, playing a vital role in the innate immune system of all living organisms [11], [12]. These peptides can identify and disrupt foreign lipid patterns in membranes without harming healthy cells [13]. Notably, AMPs often possess shared properties: they typically consist of 10–40 residues, adopt an α-helix secondary structure when they interact with membranes, maintain a positive net charge and contain a significant number of non-polar residues. These features, along with their pronounced transversal hydrophobic moment in their folded state, facilitate interactions with lipid bilayers. Thus, the use of these molecules as therapeutic agents is an obvious lesson from Nature that can be exploited to design new antibiotics, anticancer or antiaging drugs [10]. More than 3000 AMPs have been identified from a range of sources such as animals, insects, plants, and bacteria [14]. These peptides are effective against both Gram-positive and Gram-negative bacteria, and many also exhibit anticancer and antiviral properties [15]. Recent research in different animal models suggests that AMPs may have applications beyond combating microbial infections and cancer, particularly in addressing aging and diseases related to aging[16]. Nonetheless, despite their promising potential, only a handful of these peptides are currently utilized in clinical settings [14]. A significant challenge in AMP design is the limited understanding of their interaction mechanism with pathological cell membranes as well as its relationship to their sequence and structure [17], [18], [19]. Initial efforts to develop antimicrobial peptides (AMPs) were predominantly based on trial and error, stemming from a limited comprehension of their mechanism of action [20]. This highlights the importance of adopting methods that utilize quantitative criteria or are grounded in rational design [21]. In particular, their selectivity for different membrane compositions, action mechanism and effectiveness should be characterized if we aim to take advantage of these interesting molecular structures.

To fully understand and utilize the therapeutic potential of AMPs, it is crucial to delve into the intricacies of their interactions with cell membranes. The use of high-resolution wet-lab methods, such as X-ray crystallography or NMR spectroscopy, to these systems is a challenge due to the presence of lipids, difficulties to crystalize, and eventual conformational degeneration among other factors. In general, determining structures of peptides and proteins in membranes is complex since experimental conditions on which they depend are difficult to control and often diverge from physiological settings. Molecular Dynamics (MD) simulations emerge as a powerful tool for studying biological and physical systems at the atomic level, especially with advancements in computational resources and novel algorithms [22]. Even though these simulations reproduce just a virtual and idealized model of the real world, their description of the studied systems is highly detailed and they provide a great deal of information at different levels including structural, mechanistic, energetic, thermodynamic, dynamic and even kinetic, that justifies the approach. Therefore, simulation techniques can contribute to advance our understanding of the properties of AMPs.

Despite their power and precision, MD simulations, particularly unbiased MD simulations - which are simulations without any pre-set biasing or steering forces - have inherent limitations in their ability to explore conformational configurations of complex systems. One of the primary constraints is the simulation time required to capture rare events or transitions between states, as they might demand timescales far longer than those accessible by conventional MD simulations using reasonable computational resources. In systems with multiple energetic minima, the simulation may become trapped in one of these minima without transitioning to other relevant states. This difficulty in overcoming energy barriers and the lack of adequate sampling underscore the need for biased simulation methods [23]. In these latter approaches, the sampling is artificially favored along a small group of collective variables (CVs). These are functions of the atomic coordinates that act as orthogonal parameters that can capture the essential dynamics of a system, representing the configurational changes or transitions that we aim to characterize. By targeting these CVs, biased methods apply a force or potential, effectively pushing the system out of local minima and ensuring that the most relevant and often elusive regions of the system's phase space are explored. Metadynamics [23] is a well-known biased MD method able to provide in a single simulation the free energy surface (FES) as a function the selected set of CVs (potential of mean force, PMF, when only one CV is used). Metadynamics is based on the addition of an increasing bias potential to the potential energy of the simulated system, specifically at regions where the system is trapped, thus facilitating the sampling of the system through the CVs. The addition of the bias potential along the trajectory once the diffusive regime is reached allows the reconstruction of the PMF. These methods have been used to examine a variety of biological systems, including preliminary approximations that analyze the interactions of AMPs with lipid bilayers, employing diverse techniques [24], [25], [26].

In this study, we employed both unbiased MD simulations and metadynamics to investigate the interaction of renowned AMPs, specifically magainin-2 [27], pleurocidin [28], CM15[29], LL37 [30], and clavanin [31],with lipid bilayers of compositions that have been previously used as minimalist models of healthy mammals, cancer cells, and bacteria cell membranes [33], [34], [35]. These specific peptides were selected for several reasons. Firstly, they are known for their demonstrated effectiveness and wide-ranging antimicrobial/anticancer activity. Additionally, they exhibit a diversity of lengths and charges, reflecting the natural variability found in AMPs. Furthermore, their suitability for the Martini model simulations is reinforced by their documented helical structures, as evidenced in the Protein Data Bank (PDB). This aligns with the constraint of a helical structure adopted for the simulations. The specificity of the interaction of each AMP by the different bilayers was determined from the difference in the standard Gibbs energy of interaction for each case. A variety of parameters were considered in our calculations including the distance between the peptide and the membrane, the relative orientation of the peptide axis (tilt) and transversal section (spinning), the impact of the water model, the electrostatic charge of the C- and N-termini of the peptide, and also how the mobility of the lipids in the normal direction of the plane of the membrane affect the energy profiles. This detailed study was performed to discard possible artifacts in the results arising from the choice of specific simulation parameters, thus being useful as a reference for future studies of similar systems. Our results are expected to contribute to the better understanding of the interaction between AMPs and model membranes, paving the way for the optimized design of these promising peptides.

## 2 Materials and methods

### 2.1 Description of the simulated systems

**Fig. 1:**
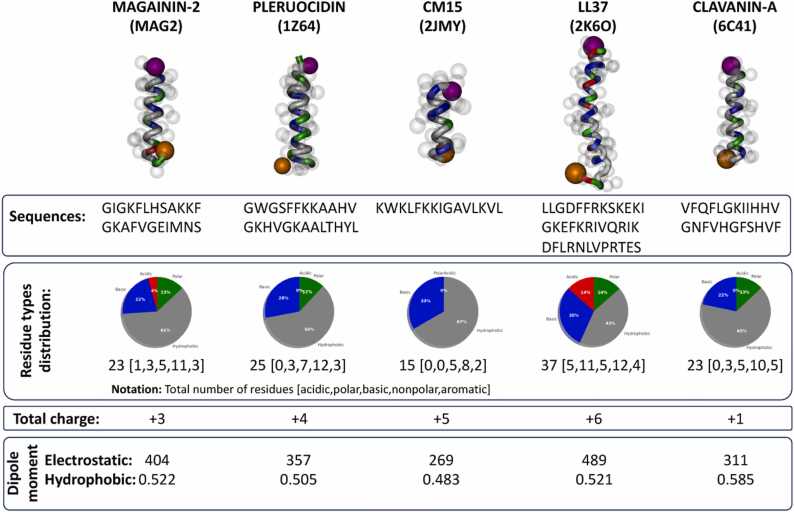
**-** Structure of the AMPs employed for the simulations together with the sequence, PDB code and the structural properties. The colors in the pie-charts represent the percentage of basic (blue), acidic (red), polar (green) and hydrophobic (grey) residues in each sequence. The units of the electrostatic dipole moment are Debyes and the hydrophobic moment was obtained from HELIQUEST [32].

The interaction between five AMPs (magainin-2, pleurocidin, CM15, LL37 and clavanin) with three model membranes was characterized by unbiased and biased MD simulations. The idealized helical structure of the employed AMPs, together with their main quantitative features, are shown in Fig. 1. Briefly, all of them are cationic, with total charge between +1 and +6, relatively short, with chain length between 15 and 37 amino acids, and with significant electrostatic and hydrophobic dipolar moments.

The features described above allow AMPs to optimize their interaction with pathogenic and pathologic membranes and, eventually, destroy them. The employed membrane models have been previously used to mimic the envelope of a mammal healthy cell (POPC:100%) [33], of a bacteria (POPE:10% and POPG:90%) [34] and of a cancer cell (CHOL:28.4%, DOPE:20%, DOPC:20%, DPSM:18.4% and DOPS:13.2%) [35]. The selection of these membrane models was based on various criteria aimed at encompassing a range of complexities from simple to more compound compositions. This strategic choice was driven by the desire not only to study a broad spectrum of lipid interactions but also to streamline the eventual laboratory preparation of liposomes, thus facilitating their examination through experimental biophysical and physico-chemical methodologies.

The simplest cell membrane model, POPC was selected for its role as one of the most abundant lipids in mammalian membranes [33]. This lipid, featuring a combination of saturated and unsaturated fatty acid chains, is extensively present in cell membrane lipid bilayers, highlighting its significance. Its prevalent use in biophysical studies as a model lipid is attributed to its ubiquitous nature and representative composition of mammalian cell membranes [33]. On the other hand, the lipid bilayer consisting of 10% POPE and 90%POPG has been previously proposed as a simplified representative for the inner membrane of bacteria based on the high fractions of these lipids on most common bacterial strains, both gram-positive and gram-negative [34]. However, the relative ratios of these lipids vary. By opting for a model with a significant charge contribution from POPG, the study aimed to assess the impact of this negatively charged lipid on peptide interactions, composed of CHOL, DOPE, DOPC, DPSM, and DOPS, aims to mimic the disrupted asymmetry characteristic of cancerous cells compared to healthy mammalian cells [35]. This model emphasizes the aberrant externalization of phosphatidylserine (PS) in cancer cells, marking a pivotal shift in membrane composition that could influence interactions with therapeutic agents targeting these cells. It is crucial to acknowledge that actual cellular membranes harbor a far more diverse array of lipids and other components, such as proteins, which play significant roles in their function and structure. However, the use of simplified membrane models presents distinct advantages. Simplified models offer a controlled environment to dissect and understand the fundamental principles of lipid interactions and the mechanisms of action of AMPs without the confounding effects of the myriad other constituents found in natural membranes. This approach allows for the isolation of specific effects and interactions, providing clearer insights into how AMPs interact with membranes of varying compositions.

### 2.2 General simulation setup and parameters

All simulations were carried out using the Martini 2.2 coarse grained (CG) force field [36], which reduces the number of particles required to represent molecules by grouping a number of heavy atoms, typically 4, in one single interaction center (bead). The starting geometry of the peptides was taken from the Protein Data Bank [37] (PDB: **2MAG**, **1Z64**, **2JMY**, **2K6O**, **6C41**). These PDB entries will be used as references to name these peptides throughout the rest of the article. Then, the *martinize.py* script [38] was employed to map from atomistic to CG resolution and get the topology, assuming they are fully helical. It should be noted that in the Martini force field the secondary structure of the peptide is restrained throughout the simulation [39] regardless the peptide is in the water bulk or interacting with the membrane. This situation is unrealistic since the peptides are expected to be folded as a helix only when they are partially embedded in the membrane. The artifact associated to this assumption will be considered later in the analysis. An additional simulation using magainin-2 without secondary structure restraints, i.e. in random coil, was performed. This additional simulation will be labelled as **2MAG-RC**. Both the standard Martini water model and the polarizable version of this solvent were employed to study the impact of this variable in all membrane models and for the five peptides employed. It is important to note that the polarizable water model requires significantly higher computational costs (approximately 2.25 times slower in our calculations) compared to the non-polarizable counterpart. Additionally, just for magainin-2 with the standard water model, 10% of antifreeze particles were added to assess how they affect the interaction between the peptide and the lipid bilayer.

In these simulations, conducted at physiological pH, the peptides were maintained in their zwitterionic form. Additionally, simulations with both the N-terminal and C-terminal ends uncharged were performed for magainin-2. This non-zwitterionic system will be labelled as **2MAG-NC** (NC being the acronym of No Charge). Furthermore, magainin-2 system was also simulated with a harmonic restrain on the *z*-coordinate of the glycerol groups of the lipids, using a force constant of *k* = 300 kJ mol^*−*1^ nm^*−*2^. These simulations will be labelled as **2MAG-RM** (RM being the acronym of Restricted Membrane). This restraint prevents strong deformations of the membrane in the *z* direction. The decrease in the number of degrees of freedom of the system associated to this restraint has already been proposed by Su *et al.* (2020) [40] in their studies of the organization of various antimicrobial peptides in membranes using unbiased MD simulations, to avoid strong deformations of the membrane model.

The structure and topologies of the membrane models were obtained from the SuPepMem database [41]. Both the peptide and the pre-equilibrated membrane model containing 250 lipids per leaflet were inserted into a virtual box with the AMP molecule at a distance larger than 4 nm from the center of the bilayer. Then, the whole system was solvated, removing the water molecules from the hydrophobic region of the membranes, and a *steepest descent* minimization was executed to avoid highly unfavorable interactions.

An NPT ensemble was employed at 1 bar and 300 K using a semi-isotropic Parrinello-Rahman barostat [42] and a V-rescale thermostat [43]. The LINCS algorithm was employed to remove bond vibrations [44]. Electrostatic interactions were calculated using reaction field with a cutoff of 1.1 nm [45]. Van der Waals interactions were calculated using also a 1.1 nm radius cutoff. Unbiased MD simulation and metadynamics production trajectories of at least 5 and 30 µs for each type of simulation, respectively, were obtained, with a time step for the integration of the motion equations of 25 fs.

The unbiased and biased (metadynamics) MD simulations were carried out with the GROMACS 2021.4 software [46] and Plumed 2.8 [47]. Molecular images were prepared using visual molecular dynamics (VMD)[48]. The analyses of the simulations and their representations were performed with Python scripts developed specifically for this purpose, based primarily on the MDAnalysis [49], Numpy [50] and Matplotlib [51] libraries.

***Analysis of the unbiased MD simulations***.

**Fig. 2:**
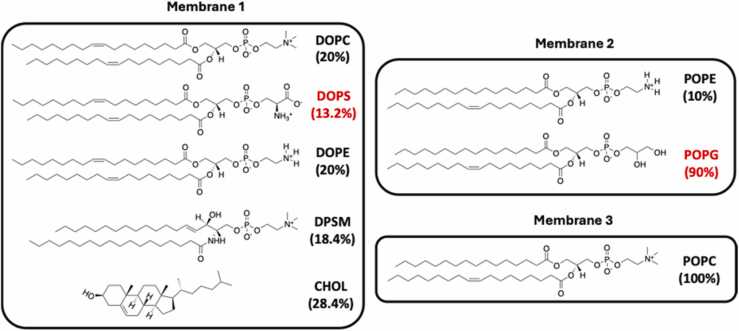
**-** Composition of the membrane models [33], [34], [35] together with the structure of the corresponding lipids. Anionic lipids are labelled in red. DOPC: 1,2-Dioleoyl-sn-glycero-3-phosphocholine; DOPS: 1,2-Dioleoyl-sn-glycero-3-phospho-*L*-serine; DOPE: 1,2-Dioleoyl-sn-glycero-3-phosphoethanolamine; DPSM: Egg sphingomyelin; CHOL: Cholesterol; POPE: 1-Palmitoyl-2-oleoyl-sn-glycero-3-phosphoethanolamine; POPG: 1-Palmitoyl-2-oleoyl-sn-glycero-3-(phospho-*rac*-(1-glycerol)); POPC: 1-palmitoyl-2-oleoyl-sn-glycero-3-phosphocholine.

All the MD simulations started with the peptide in the bulk water, without significant interaction with the membrane model. A number of properties were determined to monitor the behavior of the AMP molecule along the trajectories, including the distance to the bilayer, relative orientation, number of contacts, and diffusion parameters Fig. 2.

The tilt angle (φ) was defined as the angle between the vector joining the N- and C-termini of the peptide ($\vec{\mathrm{NC}}$) and the normal vector to the plane of the membrane (the *z* axis). Specifically,

(1) $$\cos\left(\varphi \right)=\frac{{\left(\vec{\mathrm{NC}}\right)}_{z}}{\left|\vec{\mathrm{NC}}\right|}\alpha$$

**Fig. 3:**
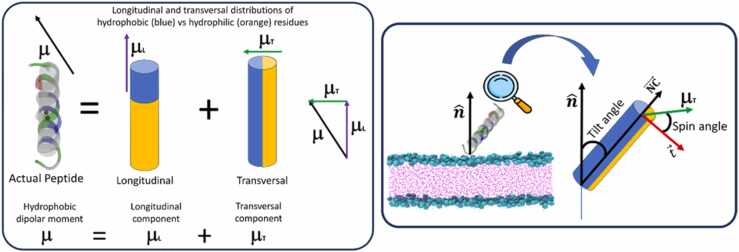
**-** Peptide hydrophobic moment and angles defining the “azimuthal rotation”. Left: Representation of the components of the hydrophobic moment, decomposing it into longitudinal and transversal distributions. Right: Depiction of the "azimuthal rotation" using the angle between two vectors related to the peptide's orientation. The "spin angle" is defined, with 180º indicating an orientation where the transverse hydrophobic dipolar moment points towards the membrane, following SuPepMem database [41] criteria.

Where ${\left(\vec{\mathrm{NC}}\right)}_{z}$ is the *z*-component of the $\vec{\mathrm{NC}}$ vector, $\left|\vec{\mathrm{NC}}\right|$ is its norm, and α takes the value −1 or +1 depending on whether the peptide is closer to the upper or lower leaflet, respectively. Thus, φ = 0° corresponds to the N-terminus approaching the membrane and φ = 180° to the C-terminus (Fig. 3).

Esteban-Martín and Salgado[52] have introduced an “azimuthal rotation” based on the angle formed by two vectors.The first vector points in the tilt direction, being perpendicular to the helix axis and lying in the plane defined by the helix and the *z*-axis. The second is a reference vector in the plane perpendicular to the helix axis, directed towards the Cα of a chosen residue. In our study, the reference vector is determined by the transversal component of the hydrophobic dipolar moment (Fig. 3), following the criteria used in the SuPepMem database [41]. This specific angle will henceforth be termed the “spin angle”. Thus, a spin angle of 180º corresponds to a helix orientation where the transversal component of the hydrophobic dipolar moment points towards the membrane. The hydrophobic dipolar moment $\vec{\mu }$ is defined as:

(2) $$\vec{\mu }=\sum \mu_{i}{\vec{r}}_{i}$$

where *μ*_*i*_ is the hydrophobic weight of the residue as defined by Fauchère et al. [53] and ${\vec{r}}_{i}$ the relative position of each backbone bead with respect to the center of mass of the peptide.

The longitudinal component of $\vec{\mu }$ is the projection of this vector on the longitudinal axis of the peptide:

(3) $${\vec{\mu }}_{L}=\left(\vec{\mu }\cdot \hat{\mathrm{NC}}\right)\hat{\mathrm{NC}}$$

where $\hat{\mathrm{NC}}$ is an unitary vector in the direction of $\vec{\mathrm{NC}}$. Then, the transversal component of $\vec{\mu }$ would be:

(4) $${\vec{\mu }}_{⊥}=\vec{\mu }-{\vec{\mu }}_{L}$$

The vector $\hat{t}$, contained in the plane formed by $\vec{\mathrm{NC}}$, and $\overset{ˆ}{n}$ which is perpendicular to $\vec{\mathrm{NC}}$, is given by:

(5) $$\hat{t}=\hat{\mathrm{NC}}\times \left(\hat{\mathrm{NC}}\times \hat{n}\right)$$

Being the spin angle:

(6) $$\xi =\arccos\left(\frac{\hat{t}\cdot {\vec{\mu }}_{⊥}}{\left|{\vec{\mu }}_{⊥}\right|}\right)$$

The result of this calculation lays between 0 and 180 degrees. In order to obtain the value of ξ in the domain [0, 2π] we calculated the cross product $\left(\hat{t}\times {\vec{\mu }}_{⊥}\right)$ and checked if it is parallel or antiparallel to $\hat{\mathrm{NC}}$ from the sign of the escalar product by this vector:

(7) $$\hat{\mathrm{NC}}\cdot (\hat{t}\times {\vec{\mu }}_{⊥})$$

which is > 0 when ξ < 180º and < 0 when ξ > 180º.

The number of contacts between the different types of peptide residue and each lipid type in the membrane as a function of time were computed employing the *gmx mindist* GROMACS tool [46] with a cutoff distance of 0.6 nm. The average values were normalized per number of residue beads of each type in the peptide in order to make comparable the results obtained for amino acids of different size and number of units of each type.

The AMP lateral displacements along the membrane using time windows of 0.2, 2 and 100 ns were calculated following the methodology described in a previous work [54]. The resulting curves follow a Rayleigh distribution [55], so they can be fitted to the two-dimensional random walk equation leading to the lateral diffusion coefficients:

(8) $$P\left(r,∆t\right)=\frac{r}{2D∆t}\exp\left(-\frac{r^{2}}{4D∆t}\right)$$

where *r* is the lateral displacement and *D* is the lateral diffusion coefficient.

Density profiles of lipid tails, lipid headgroups and peptide beads along the *z*-axis were also determined using the *density* GROMACS tool. For this calculation just the last µs of the trajectories was considered.

### 2.3 Metadynamics simulations

For the metadynamics simulations, different conditions have been essayed including CV sets, Gaussian heights and values for the biasing factor parameters in the well-tempered approach. The final set of CVs selected for production runs was formed by the following two structural parameters: (*i*) the *z* component of the vector joining the center of the AMP with the center of the bilayer; and (*ii*) the cosine of the angle formed between the symmetry axis of the helix -the vector joining connecting the C and the N-termini of the peptide- and the normal vector to the bilayer plane (i.e. the cosine of the tilt angle). Complementarily, additional simulations were performed by restraining the second CV to zero (the peptide staying perpendicular to the normal vector of the membrane plane) and using the spin angle as a CV. This new CV allows to determine how the energy profile changes when the peptide rotates around the symmetry axis of the helix, assuming that it is parallel to the membrane plane.

Convergence of the FES was achieved with the following CV parameters: Gaussians width (σ_*z*_ = 0.03 nm, σ_*cos (tilt)*_ = 0.01, σ_*spin*_ *=* 0.01 rad), Gaussian height (*W* = 2.2 kJ/mol), biasfactor (*BF* = 50); using six walkers and 25 µs long trajectories per walker. In order to optimize the calculations, the sampling was restricted to the region of interest by introducing two artificial energy barriers at *z* = 0 nm (the center of the lipid bilayer) and at *z* = 8 nm. These energy barriers are expressed as narrow harmonic potentials using the UPPER_WALLS and LOWER_WALLS Plumed functions with a force constant KAPPA= 50000 kJ·mol^−1^·nm^−2^. These energy barriers prevent the interaction of the peptide with the opposite leaflet of the membrane through the PBCs or directly crossing it. The FES from the metadynamics simulations were obtained using the *sum_hills* module[47] of Plumed. Additionally, the projection of these 2D free energy maps on each CV was obtained upon numerical integration of the probability distribution with respect to the complementary CV and transforming back the resulting integrated values into a 1D-PMF by taking their logarithm and then multiplying by the negative Boltzmann factor. This was also done by using the *sum_hills* tool. The overlap of these 1D-PMF profiles, determined every ∼200 ns were employed to check for the convergence of the Metadynamics simulations. The standard deviation of blocks of these profiles over the last 30% of the trajectories as a function of each CV was used as an estimation of the uncertainty of the 1D-PMFs.

The standard Gibbs energy for each system was determined from the numerical integration of the probability function over the FES. The derivation of the employed equation arises from the Boltzmann factor, which is proportional to the probability $p_{i}$ that a system in a certain state *i* has as a function of the corresponding energy $W_{i}$ at a given temperature:

(9) $$p_{i}\propto e^{-\beta W_{i}}$$

where $\beta ={\left(\mathrm{kT}\right)}^{-1}$, *k* is the Boltzmann constant and T the absolute temperature of the system. Then, taking the sum over all the possible states as a normalization factor $Q=\sum e^{-\beta W_{j}}$:

(10) $$p_{i}=\frac{e^{-\beta W_{i}}}{Q}$$

The adsorption of the peptide onto the surface of a lipid membrane parallel to the XY plane in an aqueous solution, with the whole system contained in a box of volume V, will be considered here. It will be assumed that the peptide is 100% helical and rigid, so it can be approached as a cylinder. The translation coordinates ($\vec{r}$) and the three Euler angles (ϒ), taking the center of the membrane as a spatial reference system, will be employed to univocally specify both the position and orientation of the peptide in the configuration space. Under these assumptions the energy for the interaction between the peptide and the membrane depends on these six degrees of freedom: W = W($\vec{r},\Upsilon$.

A partition coefficient, κ, can be defined as the ratio of the probabilities per unit volume for the peptide interacting with the membrane or free in solution (subscripts b and f, respectively), where the energy origin chosen for both states should be the same:

(11) $$\kappa =\frac{p_{b}/V_{b}}{p_{f}/V_{f}}=\frac{\frac{q_{b}}{Q\cdot V_{b}}}{\frac{q_{f}}{Q\cdot V_{f}}}=\frac{q_{b}/V_{b}}{q_{f}/V_{f}}$$

where $q_{b}$ and $q_{f}$ are the configurational partition functions for the two states of the system. Let’s assume that W depends exclusively on the coordinates *z* and φ; with z being the projection along the Z axis of the distance between the center of mass of the peptide and that of the membrane, and φ the tilt angle of the peptide. Thus, the potential of mean force (PMF), computed using these CVs, can be used as an estimation of the interaction energy. Under these assumptions, the partition function per unit volume $q_{b}/V_{b}$ can be expressed as:

(12) $$\frac{q_{b}}{V_{b}}=\frac{1}{V_{b}}\iint \mathrm{dxdy}\int_{0}^{\pi }d\theta \int_{0}^{2\pi }d\psi \int_{0}^{\lambda }\int_{0}^{\pi }e^{-\beta W\left(z,\varphi \right)}\mathrm{sen}(\varphi )\mathrm{dzd}\varphi$$

or, equivalently:

(13) $$\frac{q_{b}}{V_{b}}=\frac{4\pi^{2}}{\lambda }\int_{0}^{\lambda }\int_{1}^{-1}e^{-\beta W\left(z,\varphi \right)}\mathrm{dzdcos}(\varphi )$$

where ∬dxdy is the area of the membrane patch considered in the simulation (A), $\int_{0}^{\pi }d\theta \int_{0}^{2\pi }d\psi =4\pi^{2}$ and $V_{b}=\lambda A$, λ being the distance threshold beyond which the interaction between the peptide and the membrane is considered to be negligible. When the distance between the peptide and the membrane is larger than λ, the energy of the system does not depend anymore on *z* and φ and so, it is considered to be a constant $W^{\prime }$ that we can set to zero. Then,

(14) $$\frac{q_{f}}{V_{f}}=\frac{1}{V_{f}}e^{-\beta W^{\prime }}∭\mathrm{dxdydz}\int dϒ=8\pi^{2}e^{-\beta W^{\prime }}=8\pi^{2}$$

where $∭\mathrm{dxdydz}=V_{f}$ and $\int dϒ=8\pi^{2}$. By replacing [13] and [14] in [11]:

(15) $$\kappa =\frac{1}{2\lambda }\int_{0}^{\lambda }\int_{1}^{-1}e^{-\beta W\left(z,\varphi \right)}\mathrm{dzdcos}(\varphi )$$

The relative weight of the minimum in this integral, compared to the rest of the energy profile, is illustrated in Fig. S1.

If, additionally, it is assumed that W only depends on *z*:

(16) $$\frac{q_{b}}{V_{b}}=\frac{1}{V_{b}}\iint \mathrm{dxdy}\int dϒ\int_{0}^{\lambda }e^{-\beta W\left(z\right)}\mathrm{dz}=\frac{8\pi^{2}}{\lambda }\int_{0}^{\lambda }e^{-\beta W\left(z\right)}\mathrm{dz}$$

Resulting in:

(17) $$\kappa =\frac{1}{\lambda }\int_{0}^{\lambda }e^{-\beta W\left(z\right)}\mathrm{dz}$$

which could be directly computed from the integration of the PMF as a function of the *z* coordinate. From this expression, the Gibbs energy for the adsorption of the peptide from the aqueous solution to the membrane would be:

(18) ∆G=−kTln(κ)

Taking a cubic box with one molecule in 1660 Å^3^, free in solution or interacting with a virtual membrane, as idealized reference states for both situations, thus corresponding to the standard 1 M concentration reference state [56]:

(19) $$\kappa^{0}=\kappa \;\frac{\lambda }{V_{0}^{1/3}}$$

(20) $${∆G}^{0}=-\mathrm{kT}\ln(\kappa^{0})$$

To determine the thermodynamic feasibility of a peptide's adsorption to different membrane surfaces from an aqueous solution, we define the Gibbs free energy of adsorption for a peptide moving from water to membrane M as.

(21) ΔG^0^_water→M_ = G_M_ − G_water_

Similarly, the Gibbs free energy of adsorption from water to membrane C is expressed as.

(22) ΔG^0^_water→C_ = G_C_ − G_water_

From these expressions, the Gibbs free energy of transfer from membrane M to membrane C is the difference in the peptide's adsorption free energy to the two membranes, calculated as.

(23) ΔG^0^_M→C_ = G_C_ − G_M_

This can be reformulated using the adsorption free energies, leading to.

(24) $$\begin{array}{c} \Delta \Delta G^{0}=\Delta G_{water\to C}^{0}-\Delta G_{water\to M}^{0}\\ \Delta \Delta G^{0}=G_{C}-G_{water}-(G_{M}-G_{water})=G_{C}-G_{M}=\Delta G_{M\to C}^{0} \end{array}$$

providing a straightforward calculation for the preference of the peptide for membrane C over membrane M. This expression is interesting because as it does not rely on the artificial assumption that the peptides adopt a helical conformation in the bulk water.

## 3 Results and discussion

### 3.1 Unbiased MD simulations

**Fig. 4:**
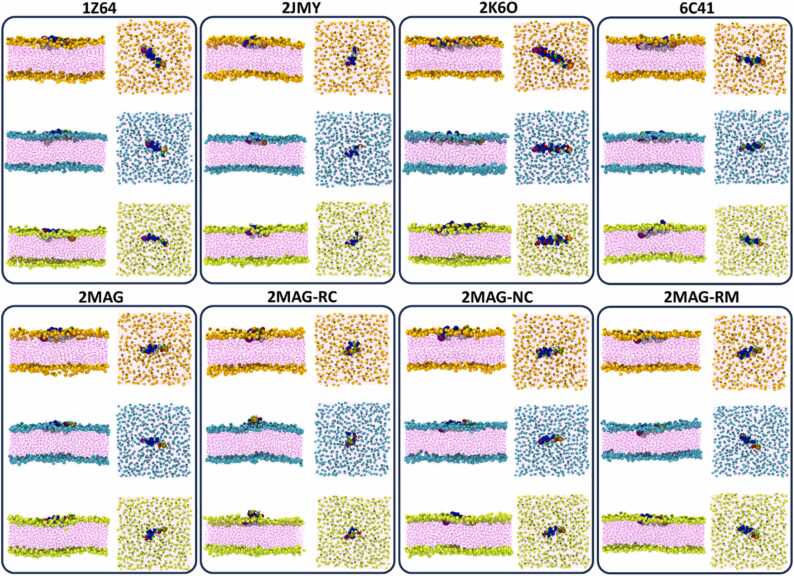
**-** Snapshots corresponding to the side and top views of the last frame (t = 5 µs) of all the unbiased MD simulations performed in this work. The name (PDB code) of the AMP employed in each trajectory, as defined in the methods section, is indicated on top of each panel. **2MAG-NC** denotes the non-zwitterionic magainin-2 system, while **2MAG-RC** signifies magainin-2 simulations without secondary structure restraints, representing a random coil conformation. Additionally, **2MAG-RM** designates a system with harmonic constraints applied to the *z*-coordinate of lipid glycerol groups. For each system the results of the simulation obtained in the membrane models 1, 2 and 3 (see compositions in) are shown with the lipid heads in orange, cyan (blue) and green colors, respectively. Waters are not shown for clarity.

**Fig. 5:**
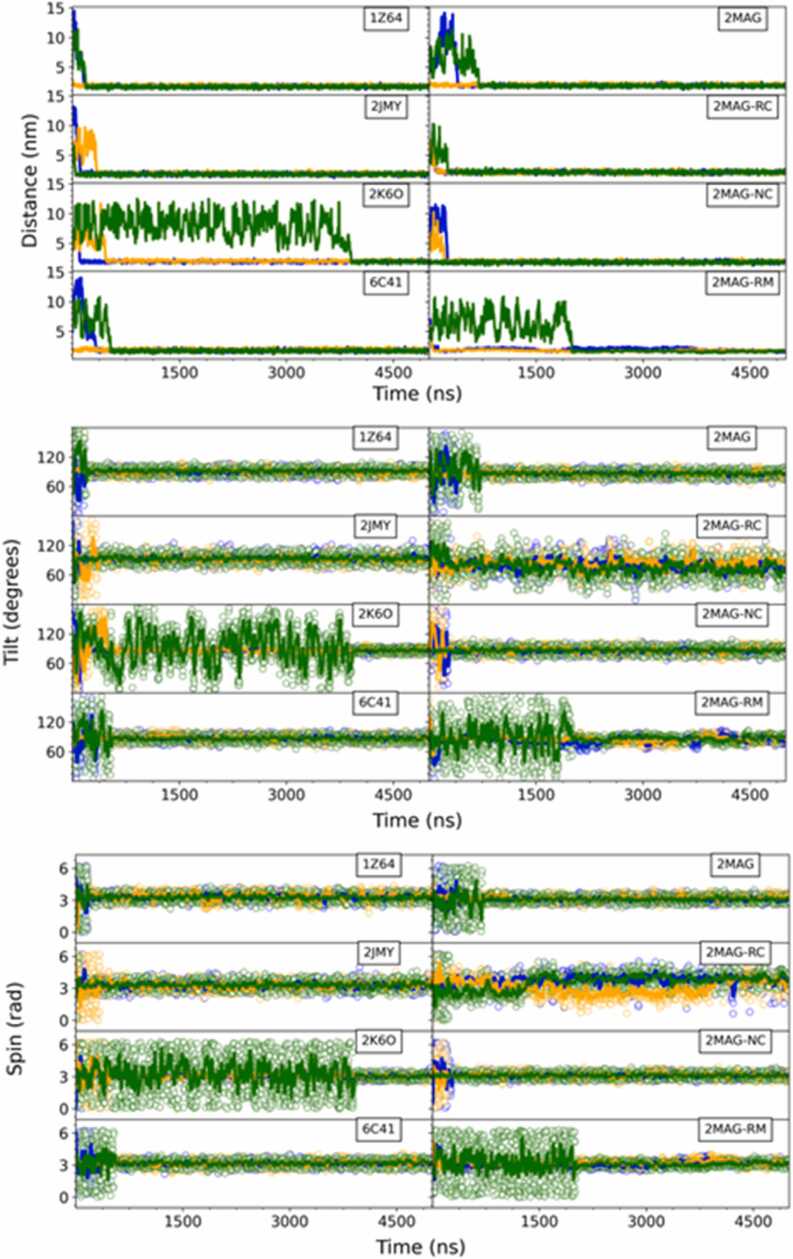
**-** Distance between the center of the membrane and the center of mass of each AMP (top) together with the tilt (middle) and spin (bottom) angles of the simulated peptides as a function of time, using unbiased MD simulations. The name (PDB code) of the AMP employed in each trajectory, as defined in the methods section, is indicated on top of each panel. **2MAG-NC** denotes the non-zwitterionic magainin-2 system, while **2MAG-RC** signifies magainin-2 simulations without secondary structure restraints, representing a random coil conformation. Additionally, **2MAG-RM** designates a system with harmonic constraints applied to the *z*-coordinate of lipid glycerol groups. For each system the results of the simulation obtained in the three membrane models are shown with the lipid heads in orange, cyan (blue) and green colors, for membrane 1, 2 and 3, respectively, as labeled in Fig. 2.

All the individual AMPs (**2MAG**, **1Z64**, **2JMY, 2K6O, 6C41**, **2MAG-RC**, **2MAG-NC** and **2MAG-RM**) were initially located at a relatively large distance from the bilayer surface, in such a way that both structures (the peptide and the membrane) did not interact with each other. The peptide spontaneously diffused, eventually reaching the membrane model. Once the AMP met the bilayer, it did not go back to the water bulk anymore and integrated itself into the lipid assembly (Fig. 4 and top panel of Fig. 5).

The interaction between the AMP and the bilayer strongly influenced both the tilt and spin angles of the peptide. Specifically, the peptide aligned itself parallel to the plane of the membrane, resulting in a tilt angle of approximately 90 degrees. Additionally, the transverse hydrophobic moment of the peptide pointed towards the hydrophobic core of the lipid bilayer, resulting in a spin angle of approximately 180 degrees (as shown in Fig. 5, in the middle and bottom panels). It is worth noting that this behavior was consistent across all cases except for the **2MAG-RC** system, where the definition of both angles is ambiguous due to the lack of forced helicity in the peptide. The qualitative behavior of all the systems was equivalent, although small quantitative differences could be observed (see Tables S1-S3). It is interesting to note that for all the AMPs, the final distance to the center of the bilayer was consistently shorter when using the POPC membrane model compared to the simulations involving the more complex membrane models. The difference in the distance between the pathogenic membrane models were significant for all the peptides except for the different variants of **2MAG**. The largest difference corresponded to clavanin (**6C41**) which has the lowest electrostatic charge (+1). For all these systems, the penetration was slightly deeper for the membrane model 2 than for the membrane model 1 (Table S1). Interestingly, the approach to the lipid surface was notably slower when using the pure POPC lipid bilayer as compared to the more complex membrane models. However, the final distance to the center of the bilayer was slightly shorter for the pure POPC bilayer than for the other two membrane types. Moreover, the tilt and spin angles exhibited similar patterns across all the systems. The slower approach to the POPC lipid bilayer could be attributed to the lack of electrostatic attraction in that case, since the single compound of this membrane model is neutral. Despite some slight differences in average final angles for each peptide (as detailed in Tables S2 and S3), neither the tilt nor the spin angles displayed clear or systematic variations for the same peptide among the different membrane models. These variations cannot be solely explained by differences in the length of the various AMP molecules, which obviously affect the position of their center of mass. For instance, the sequence of **2JMY** has fewer than half the number of residues compared to **2K6O** (15 vs. 37), yet their final distances from the bilayer center are similar. Additionally, the total charge of both molecules is similar (5 vs. 6), although the charge density is significantly higher for the shorter peptide. Notably, there was an almost ∼16-degree difference in the tilt angle between these two structures.

**Fig. 6:**
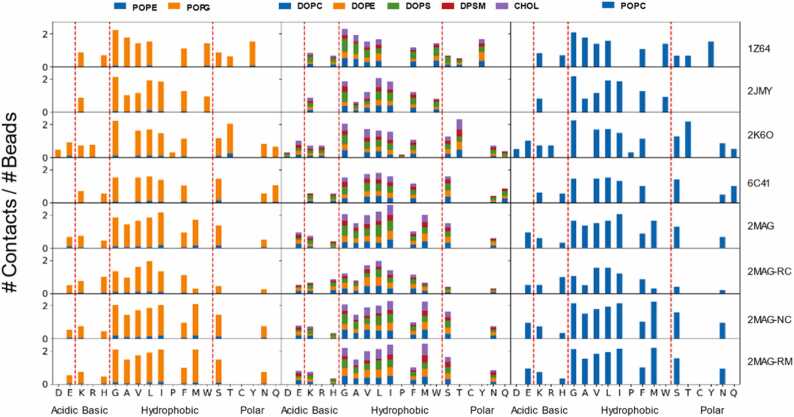
- Average number of contacts between each type of residue and the different lipid types (normalized by total number of beads of the amino acid), averaged over the last microsecond of all the unbiased trajectories and using a cut-off of 0.6 nm. Each row corresponds to a distinct AMP, identified by its PDB code. **2MAG-NC** denotes the non-zwitterionic magainin-2 system, while **2MAG-RC** signifies magainin-2 simulations without secondary structure restraints, representing a random coil conformation. Additionally, **2MAG-RM** designates a system with harmonic constraints applied to the z-coordinate of lipid glycerol groups. The results for each membrane model are in a different column (membranes 2, 1 and 3 in columns 1, 2 and 3, respectively) with the associated lipids represented by the colors indicated in the top legends.

The average number of contacts between each type of amino acid (normalized by the total number of beads in the peptide) and each lipid type was determined using the last microsecond of each trajectory (Fig. 6). In general, hydrophobic residues exhibited the largest number of interactions with lipids for all peptides and membrane models. Polar amino acids also displayed significant interactions across all categories, while the interaction with acidic and basic residues seemed to be weaker. It is important to note that these results were independent on the number of amino acids of each type present in the peptides, which indeed was larger for hydrophobic than for other types, due to the normalization of the count. Actually, the percentage of hydrophobic residues oscillates between 60% and 67% for all peptides except for **2K6O**, which contains 43.2% of hydrophobic residues (Fig. 1). The percentage of polar residues varies between 12% − 13% for all peptides except for **2JMY**, which does not contain any amino acid of this type. However, when serine (S) or threonine (T) are present, the number of contacts per bead for these amino acids is comparable to the average number of contacts of the hydrophobic residues. This does not happen for the acidic or basic amino acids in any case. Interestingly, there was no significant dependence on the membrane composition regarding the total number of contacts per type of residue. The differences in the contacts per type of lipid are roughly proportional to their concentration in the bilayer. Thus, each peptide exhibited a unique fingerprint for the number of contacts per type of residue which does not significantly depends on the membrane model. This coordination sphere, reminiscent of the arrangement of water molecules around an ion, is shaped by the local environment. It selectively attracts and forms interactions with specific constituents until the coordination sphere becomes fully saturated with contacts. These interactions may encompass lipids of specific types, water molecules, or other molecules, if present in the simulation, depending on their respective higher affinities. This was observed when the secondary structure of the AMP was restrained, however for the simulation **2MAG-RC** the pattern was different, as expected, due to the ambiguous structure of the macromolecule in this simulation.

While the values obtained from our unbiased simulations should not be directly compared to experimental diffusion coefficient measurements, the simulations are sensitive to both the peptide's sequence and the composition of the bilayer, thus they offer valuable insights into the behavior of AMP molecules within various membrane models.

The obtained diffusion coefficients from lateral displacements clearly exhibited a dependency on the selected time window employed for calculations. This observation suggests that the calculations were not conducted within a stationary regime, indicating that the dominant diffusive regime typically observed in experiments is not prevalent in our simulations. Nevertheless, the simulations offer valuable insights into the behavior of AMP molecules within diverse membrane models due to their sensitivity to both the peptide's sequence and the composition of the bilayer. The diffusion coefficients obtained from the lateral displacements for short time windows (0.2 and 2 ns), where Brownian diffusion is expected to dominate, were significantly lower for the membrane 1 than for membranes 2 and 3 (Figs. S2–3 and Table S4). For longer time windows (100 ns) no clear trends were observed. For a time window of 0.2 ns the values obtained for the membranes 2 and 3 were similar to each other. The differences were more significant at 2 ns, with larger values in the membrane model 2 than in the model 3 except for **2MAG-NC** and **2MAG-RM**. For the largest time window, the trend was similar but the diffusion coefficient in the membrane model 1 is, in general, comparable to that in the other two bilayers, and even higher in **2JMY** and in **2MAG-RM**. There was also a systematic decrease in the diffusion coefficient when increasing the length of the time window employed for the calculation, the maxima taking values of ∼12·10^−7^ cm^2^/s at 0.2 ns for **2MAG-RC**, ∼6·10^−7^ cm^2^/s at 2 ns and ∼0.9·10^−7^ cm^2^/s at 100 ns for **1Z64**. Additionally, the differences between the different AMP molecules were larger at short time windows. In general, it seems that the shortest time window is more sensitive to both the sequence of the peptide and the membrane composition. The physical interpretation of this is not clear, since the membrane model 2 contains the largest density of charge, that could speed down the diffusion of the peptide, and it was previously observed that the penetration of the peptide is significantly deeper in the pure POPC lipid bilayer model than in the other two membranes, what could also inhibit the diffusion of the peptide. Moreover, the shortest time scale used for these measurements does not consider large diffusive displacements but mostly short movements highly conditioned by local interactions. Note that the maxima of the displacement distributions at 0.2 ns was at ∼1 nm for all the peptides, which is comparable to the diameter of an α-helix.

In summary, the analysis of the unbiased MD simulations suggested that once the peptides encountered the various membrane models through passive diffusion, the interactions established between them were both stable and specific. The kinetics of this encounter seemed to be accelerated by long-range electrostatic interactions. Meanwhile, the internalization of the peptide, as well as its tilt and spin orientation, were consistently determined by the electrostatic and hydrophobic dipolar moments, with particular emphasis on the transversal component of the latter, as indicated by the final tilt and spin angles. Interestingly, each peptide seemed to exhibit a characteristic fingerprint in the normalized number of contacts per type of residue which did not depend on the membrane composition. The lateral diffusion coefficients of the peptides in the different membrane models, when determined using short time windows, were highly sensitive to the sequence and the bilayer composition. Notably, these coefficients yielded considerably shorter values for the membrane model 1 compared to the membrane models 2 and 3. Conversely, the two latter bilayers did not exhibit clear trends in the differences between them. However, when considering the results obtained for the largest time window, where the peptide displacement was more significant and so the diffusion was closer to the stationary regime, sensitivity to both the peptide sequence and bilayer composition was less pronounced.

### 3.2 Enhanced sampling methods

**Fig. 7:**
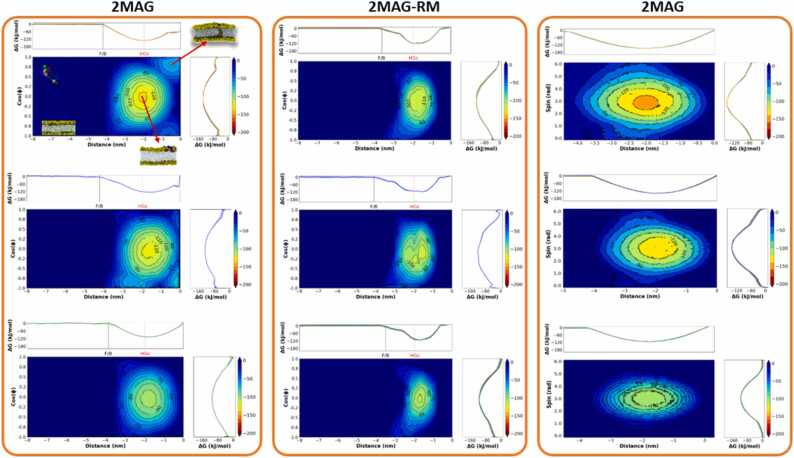
**-** Two-dimensional potential of mean force (2D-PMF) profiles for the **2MAG** system derived from Metadynamics simulations using the center of mass distance between the peptide and the lipid bilayer center (*z*) and the cosine of the tilt angle (left and middle columns) or the spin angle and *z* (right column) as CVs. The middle column simulations were performed with *z*-coordinate restraints on the glycerol groups of the lipids (**2MAG-RM**), while the simulations in the left and right columns had no lipid restraints. Results for the membrane models 1 (top row), 2 (central row) and 3 (bottom row) are presented. The colormap represents the Gibbs energy values. Projections of the PMF onto each CV (see methods section) are displayed above and to the right of the colormaps. The dotted red line in the *z*-projection indicates the position of the membranes phosphorous atoms while the dashed black line denotes the distance where the interaction between the AMP and the membrane becomes negligible. '“F/B” labels correspond to “free” and “bound” regions, respectively). Depictions in the upper left corners illustrate the peptide's representative location and orientation relative to the lipid bilayer at two local energy minima.

To get a precise quantification of the specificity of the interaction between different peptides and various membrane models, we turned to biased MD (metadynamics) simulations, aimed to identify the most stable peptide conformations within each membrane model and determine the Gibbs energy associated with the interaction between each AMP and the lipid bilayers. Metadynamics simulations were performed using two CVs, namely the cosine of the tilt angle or the spin angle, and the distance between the peptides center of mass of and the membranes center. Simulations using the cosine of the tilt angle (Fig. 3) were performed for all the systems, while those using the spin angle were specifically performed for **2MAG** and for **6C41**. In all cases the interaction energy between the peptide and the membrane was found to be favorable, comparable to the interaction between the peptide and the solvent, with a minimum lower than − 100 kJ/mol. The notably low interaction energy observed may stem from the assumption that the peptide adopts a helical conformation in bulk water—an assumption which may not fully align with the peptide's behavior, hence contributing an unexpectedly high energy penalty for the peptide’s presence in the aqueous phase. Although the total ∆G^0^ values obtained for the peptide adsorption onto the membrane appear unrealistic, relative values among different membrane models are meaningful. Specifically, they aid in determining the Gibbs energy of transfer from one membrane to another (∆_transfer_∆_adsorption_G^0^ = ∆∆G^0^) [Eq. 24]. Moreover, the energy profiles and the structures corresponding to the minima provide valuable insights into the interaction mechanism between AMPs and lipid bilayers (Fig. 7). This information encompasses the preferred location and orientation of the system's most favorable configuration, the optimal peptide orientation during its approach to the membrane model, and ultimately, the energy required for the peptide to translocate through the lipid bilayer. Across all the simulations, the global energy minima consistently occurred when the peptide was aligned parallel to the plane of the membrane, intimately integrated with the lipid headgroups and with the transverse hydrophobic dipolar moment oriented towards the bilayers center (Fig. 7 and S4-S10). Interestingly, in several systems the peptide approached the lipid bilayers with its N-terminal oriented towards the membrane surface (cosine of the tilt angle ∼−1). This orientation was particularly evident in cases such as **2MAG** and **2K6O** in the membrane model 2 (as shown in Fig. 7 and S6, respectively).

In general, the results obtained from Metadynamics align well with those observed from the unbiased MD simulations (Fig. 4, Fig. 5). However, the biased calculations offer a more comprehensive insight and guarantee that the observed results are not attributed to kinetically trapped states. Given the significant deformation of the bilayer in the presence of the AMP, metadynamics simulations with restrained *z*-coordinates of the glycerol groups of the lipids were performed for magainin-2 (**2MAG-RM**) (Fig. 7). While this additional restraint didn´t significantly affect the depth of the energy minima, it brought about notable changes in the shape of the FES. This change included the appearance of a shoulder, which likely resulted from the overlay of an energy barrier associated with the peptide's entry into the restrained bilayer surface with a relatively broad minimum.

**Fig. 8:**
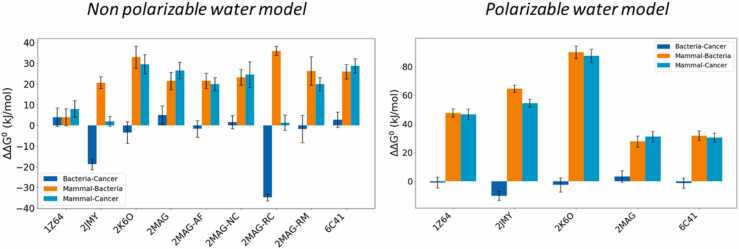
**-** Standard energy of transfer (ΔΔG^0^) of the peptides between different membrane models, as indicated in the plots, for the simulations in non-polarizable and in polarizable water model. The plotted numbers are obtained from Table S5.

To assess the impact of electrostatic polarization, metadynamics simulations were also conducted using polarizable water models for most of the systems (**2MAG**, **1Z64**, **2JMY, 2K6O, 6C41**). Additionally, simulations incorporating 10% of antifreeze particles in non-polarizable water were also performed. The standard free energies of adsorption from the water bulk to the membrane surface are shown in Table S5. From this information, the ∆∆G^0^ values, which denotes the free energy differential when a peptide's binding shifts not just from the pure POPC lipid bilayer to the more complex membrane models, but also between membranes 1 and 2, were calculated (Fig. 8). The ΔΔG^0^ from the monocomponent lipid bilayer to the multicomponent membrane models is favorable and relatively large in all simulations involving non-polarizable water but becomes even more pronounced when polarizable water is employed. The energy variances when comparing membrane models 1 and 2 are generally modest, with most falling within the uncertainty range. However, there are exceptions, specifically with peptides **2JMY** (the shortest peptide) and **2MAG-RC** (the magainin-2 structure without secondary structure constraints), which have a large preference for the membrane model 1 (Fig. 8).

Peptide **2K6O** (LL37), the largest among those examined, exhibited the most significant specificity towards pathogenic membranes in both water models. In contrast, peptide **1Z64** showed minimal specificity when in non-polarizable water. The effect of the polarizable water model on the ΔG^0^ values for peptide binding to membranes was particularly noticeable for the pure POPC lipid bilayer, with the most pronounced change observed for **2JMY**, where ΔG^0^ shifted from −71.2 to −22.1 kJ/mol. In the case of peptides like **6C41** and **2MAG**, the choice of water model had a negligible impact. Although experimental data to validate the accuracy of the polarizable water model over the non-polarizable one is not available, the differences in results are significant enough to merit serious consideration of the polarizable model's implications for peptide-membrane interaction studies, a conclusion recently supported by Risselada and colleagues [57].

The impact of the antifreeze particles in the non-polarizable water model was negligible, so they prevent the artificial crystallization of this imperfect solvent without affecting the energy of interaction with the membrane model. The simulations with **2MAG-NC** yielded results that closely resemble those obtained with the zwitterionic form of this peptide, indicating that the electrostatic state of the termini does not significantly impact the ∆G^0^ values.

What truly emerges from our study as a crucial factor in these results is the imposition of restraints on the secondary structure of the peptide, as demonstrated in the **2MAG-RC** system. Ideally, simulations employing an atomistic force field capable of not only considering the relative angles and distances between the peptide and the membrane but also the folding state of the AMP would be most desirable. However, achieving this goal proves challenging with the current computational resources, although efforts are underway to explore this possibility [58], [59], [60].

Metadynamics simulations were also employed to determine how peptide orientation affected interactions with membranes, using the spin angle—which assesses the orientation of the peptide's transversal component of the hydrophobic dipolar moment in relation to the membrane's normal—as a CV (see Methods). These simulations revealed that the peptide's most stable state was achieved when such component of the hydrophobic dipolar moment was aligned perpendicular to the membrane surface. (Fig. 7 and S10). This observation is consistent with results from unbiased MD simulations (see Fig. 5), with the additional advantage of being able to quantify the Gibbs energy changes during peptide rotation. The metadynamics FES underscores the importance of the spin angle for peptide stability, particularly when the peptide is optimally positioned in the lipid bilayer relative to the bilayer's central axis (Fig. 6).

## 4 Conclusion

We have carried out a comprehensive study of the interactions between renowned AMPs—specifically magainin-2, pleurocidin, CM15, LL37, and clavanin—and lipid bilayers that have been claimed to represent healthy mammal, cancerous, and bacterial cell membranes. Through a series of unbiased and biased molecular dynamics simulations (well-tempered metadynamics using different CV sets, water models and restraint conditions for both the peptides and the different lipid bilayers), we shed light on the intricate mechanisms by which these peptides interact with pathogenic lipid patterns.

The specificity of the interactions between the AMPs and the membranes was quantified by the differences in the standard Gibbs energy of interaction, unveiling the peptides' selective affinity towards distinct membrane compositions. In particular, it is shown that all the peptides are able to recognize multicomponent membranes (previously proposed as minimalist cancer and bacteria models), interacting with them significantly stronger than with pure POPC lipid bilayers. This specificity is critical for ensuring therapeutic efficacy while minimizing harm to healthy cells. In particular, LL37 (**2K6O**), the largest AMP analyzed, exhibited the highest specificity for membrane models 1 and 2, suggesting its superior potential for targeting disease-related cells while sparing healthy mammalian cells. Our simulations illuminated the mechanism by which AMPs approach lipid bilayers, engaging at varying depths and orientations relative to the lipid headgroups. From simulations of **2MAG-RC** in the absence of helical restraints, it became evident that the secondary structure of peptides plays a pivotal role in these interactions. On the technical side, we found that the impact of the water model in the simulations is non-negligible. The use of polarizable water models altered the standard Gibbs energy of adsorption to the membrane, especially notable in the case of **2JMY**, where the ΔG^0^ value shifted dramatically from −71.2 to −22.1 kJ/mol. This shift underlines the importance of experimental data supporting the use of the most computationally expensive model. Furthermore, the introduction of extra restraints on lipid headgroups affected the energy profiles of AMP interactions with the bilayer. The simulations with **2MAG-RM**, with the *z*-coordinates of the glycerol groups of the lipids restrained, showed that while the depth of energy minima remained largely unchanged, the shape of the potential of mean force (PMF) profiles exhibited significant alterations, indicating an additional energy barrier for peptide entry into the bilayer surface.

In general, our findings highlight the peptides' ability to integrate into lipid assemblies upon contact, with their kinetic behavior and orientation being influenced by both electrostatic and hydrophobic interactions. The optimal configuration of the interaction places the peptide parallel to the membrane surface at slightly different depths, depending on the bilayer composition. The transversal component of the peptide's hydrophobic dipole moment proved to be of paramount importance for the interaction with the membranes, determining the orientation of the molecule. It is worth emphasizing that while amphipathicity, characterized by the peptide's distinct hydrophobic and hydrophilic regions, is crucial for initiating this interaction, the transverse hydrophobic dipole moment further refines our understanding of how these peptides embed themselves into and disrupt the membrane. This nuanced view adds depth to the recognized roles of charge distribution and amphipathicity [61], [62] illustrating how the orientation and insertion dynamics provided by the transverse hydrophobic dipole moment complement these well-established mechanisms. The Gibbs energy resulted to be extremely sensitive to the rotation of the peptide around the symmetry axis of the helix. This agrees with the spin angle spontaneously obtained from the unbiased MD simulations once the peptide gets in contact with the membrane and the systems reaches the equilibrium.

The kinetics, preferred relative orientation of the peptide when approaching the membrane model, and lateral diffusion at short time scales also depends on the lipid composition of the bilayer. For instance, **2MAG** and **2K6O** clearly prefer to approach the membrane model 2 with the N-terminal oriented towards the bilayer surface, while this effect is less clear for other combinations of peptides and membrane compositions. This is probably due to the electrostatic forces that dominate the interaction at long distances. The lateral diffusion coefficients calculated using time windows of just 200 ps are significantly lower for the AMPs in membrane model 1 than in lipid bilayers of other compositions. Lateral diffusion coefficients at larger time windows are much less specific to distinguish both peptide sequences and lipid compositions.

Central to our findings is the generation of comprehensive datasets that detail these biophysical interactions. These datasets are poised to significantly impact the field of ML within bioinformatics and computational biology. The quantitative insights, such as differences in the standard Gibbs energy of interaction, peptide orientation, depth of insertion, and lateral diffusion coefficients, are invaluable for training ML models. These models can exploit our data to predict the behavior and efficacy of untested AMP sequences, thereby accelerating the design of novel peptides with optimized specificity and potency. Particularly, the free energy surfaces (FES) and potential of mean force (PMF) profiles derived from our simulations offer a rich source of data for ML algorithms to uncover underlying patterns and principles governing peptide-membrane interactions. Furthermore, the novelty of our study lies in the depth of analysis and the specific findings related to the transversal component of the peptide's hydrophobic dipole moment and its critical role in membrane interaction. While the significance of charge distribution and amphipathicity is well-established, our work quantifies the contribution of these molecular characteristics, providing a new level of insight that ML models could exploit.

Thus, our research provides a blueprint for subsequent ML applications, where our simulation data can train models to predict the behavior of novel AMPs. This predictive power could significantly reduce the time and cost associated with the experimental development of new therapeutic peptides.

Looking forward, we acknowledge the need to evolve our models to include more complex lipid compositions, such as anionic gangliosides, cholesterol, and the peptidoglycan cell wall, to mirror real biological membranes more closely. This evolution will enrich the training datasets for ML algorithms, allowing for an even more accurate prediction of AMP performance.

## CRediT authorship contribution statement

**Daniel Conde-Torres:** Writing – review & editing, Writing – original draft, Visualization, Validation, Methodology, Investigation. **Martín Calvelo:** Writing – review & editing, Writing – original draft, Supervision. **Carme Rovira:** Writing – review & editing, Supervision. **Ángel Piñeiro:** Writing – review & editing, Writing – original draft, Supervision, Methodology, Investigation, Funding acquisition, Conceptualization. **Rebeca Garcia Fandino:** Writing – review & editing, Writing – original draft, Supervision, Investigation, Funding acquisition, Conceptualization.

## Declaration of Competing Interest

The authors declare that they have no known competing financial interests or personal relationships that could have appeared to influence the work reported in this paper.

## References

[1] Gould S.B. (2018). Membranes and evolution. Curr Biol.

[2] Harayama T., Riezman H. (2018). Understanding the diversity of membrane lipid composition. Nature Reviews Molecular Cell Biology.

[3] Khan M., Lin J., Liao G., Tian Y., Liang Y., Li R. (2019). ALK inhibitors in the treatment of ALK positive NSCLC. Front Oncol.

[4] Bottazzi B., Riboli E., Mantovani A. (2018). Aging, inflammation and cancer. Semin Immunol.

[5] Garcia-Fandino R., Piñeiro Á. (2021). Delving Into the origin of destructive inflammation in COVID-19: a betrayal of natural host defense peptides?. Front Immunol.

[6] Johnson A.A., Stolzing A. (2019). The role of lipid metabolism in aging, lifespan regulation, and age-related disease. Aging Cell.

[7] Azevedo M.M., Pina-Vaz C., Baltazar F. (2020). Microbes and cancer: friends or faux?. Int J Mol Sci.

[8] Vedham V., Divi R.L., Starks V.L., Verma M. (2014). Multiple infections and cancer: implications in epidemiology.

[9] Furman D., Campisi J., Verdin E., Carrera-Bastos P., Targ S., Franceschi C. (2019). Chronic inflammation in the etiology of disease across the life span. Nature Medicine.

[10] Conde-Torres D., Blanco-González A., Seco-González A., Suárez-Lestón F., Cabezón A., Antelo-Riveiro P. (2024). Unraveling lipid and inflammation interplay in cancer, aging and infection for novel theranostic approaches. Front Immunol.

[11] Magana M., Pushpanathan M., Santos A.L., Leanse L., Fernandez M., Ioannidis A. (2020). The value of antimicrobial peptides in the age of resistance. Lancet Infect Dis.

[12] Huan Y., Kong Q., Mou H., Yi H. (2020). Antimicrobial peptides: classification, design, application and research progress in multiple fields. Front Microbiol.

[13] Claro B., González-Freire E., Calvelo M., Bessa L.J., Goormaghtigh E., Amorín M. (2020). Membrane targeting antimicrobial cyclic peptide nanotubes – an experimental and computational study. Colloids Surf B Biointerfaces.

[14] Dijksteel G.S., Ulrich M.M.W., Middelkoop E., Boekema B.K.H.L. (2021). Review: lessons learned from clinical trials using antimicrobial peptides (AMPs). Front Microbiol.

[15] Yang M., Liu S., Zhang C. (2023). Antimicrobial peptides with antiviral and anticancer properties and their modification and nanodelivery systems. Curr Res Biotechnol.

[16] Hanson M.A., Lemaitre B. (2023). Antimicrobial peptides do not directly contribute to aging in Drosophila, but improve lifespan by preventing dysbiosis. Dis Model Mech.

[17] Wimley W.C. (2010). Describing the mechanism of antimicrobial peptide action with the interfacial activity model. ACS Chem Biol.

[18] Zhang Q.Y., Yan Z.Bin, Meng Y.M., Hong X.Y., Shao G., Ma J.J. (2021). Antimicrobial peptides: mechanism of action, activity and clinical potential. Mil Med Res 2021.

[19] Chen N., Jiang C. (2023). Antimicrobial peptides: structure, mechanism, and modification. Eur J Med Chem.

[20] Epand R.M., Vogel H.J. (1999). Diversity of antimicrobial peptides and their mechanisms of action. Biochim Et Biophys Acta (BBA) - Biomembr.

[21] Li J., Koh J.J., Liu S., Lakshminarayanan R., Verma C.S., Beuerman R.W. (2017). Membrane Active Antimicrobial Peptides: Translating Mechanistic Insights to Design. Front Neurosci.

[22] Palmer N., Maasch J.R.M.A., Torres M.D.T., De La Fuente-Nunez C. (2021). Molecular dynamics for antimicrobial peptide discovery. Infect Immun.

[23] Laio A., Parrinello M. (2002). Escaping free-energy minima. Proc Natl Acad Sci USA.

[24] Hanmandlu C., Singh A., Karunakara M., Boopathi A., Wang F., Cao Y. (2016). The effect of orientation dynamics in melittin as antimicrobial peptide in lipid bilayer calculated by free energy method. J Phys Conf Ser.

[25] Simcock P.W., Bublitz M., Cipcigan F., Ryadnov M.G., Crain J., Stansfeld P.J. (2021). Membrane binding of antimicrobial peptides is modulated by lipid charge modification. J Chem Theory Comput.

[26] Kabelka I., Brožek R., Vácha R. (2021). Selecting collective variables and free-energy methods for peptide translocation across membranes. J Chem Inf Model.

[27] Gesell J., Zasloff M., Opella S.J. (1997). Two-dimensional 1H NMR experiments show that the 23-residue magainin antibiotic peptide is an alpha-helix in dodecylphosphocholine micelles, sodium dodecylsulfate micelles, and trifluoroethanol/water solution. J Biomol NMR.

[28] Syvitski R.T., Burton I., Mattatall N.R., Douglas S.E., Jakeman D.L. (2005). Structural characterization of the antimicrobial peptide pleurocidin from winter flounder. Biochemistry.

[29] Andreu D., Ubach J., Boman A., Wåhlin B., Wade D., Merrifield R.B. (1992). Shortened cecropin A-melittin hybrids Significant size reduction retains potent antibiotic activity. FEBS Lett.

[30] Bandurska K., Berdowska A., Barczyńska-Felusiak R., Krupa P. (2015). Unique features of human cathelicidin LL-37. Biofactors.

[31] Silva O.N., Fensterseifer I.C.M., Rodrigues E.A., Holanda H.H.S., Novaes N.R.F., Cunha J.P.A. (2015). Clavanin A improves outcome of complications from different bacterial infections. Antimicrob Agents Chemother.

[32] Gautier R., Douguet D., Antonny B., Drin G. (2008). HELIQUEST: a web server to screen sequences with specific alpha-helical properties. Bioinformatics.

[33] Luchini A., Vitiello G. (2021). Mimicking the Mammalian Plasma Membrane: An Overview of Lipid Membrane Models for Biophysical Studies. Biomimetics.

[34] Krok E., Stephan M., Dimova R., Piatkowski L. (2023). Tunable biomimetic bacterial membranes from binary and ternary lipid mixtures and their application in antimicrobial testing. Biochim Et Biophys Acta (BBA) - Biomembr.

[35] Nguyen H.L., Man V.H., Li M.S., Derreumaux P., Wang J., Nguyen P.H. (2022). Elastic moduli of normal and cancer cell membranes revealed by molecular dynamics simulations. Phys Chem Chem Phys.

[36] Marrink S.J., Risselada H.J., Yefimov S., Tieleman D.P., De Vries A.H. (2007). The Martini Force Field: Coarse Grained Model for Biomolecular Simulations. J Phys Chem B.

[37] Berman H.M., Westbrook J., Feng Z., Gilliland G., Bhat T.N., Weissig H. (2000). The Protein Data Bank. Nucleic Acids Res.

[38] De Jong D.H., Singh G., Bennett W.F.D., Arnarez C., Wassenaar T.A., Schäfer L.V. (2013). Improved parameters for the Martini coarse-grained protein force field. J Chem Theory Comput.

[39] Sharma P., Desikan R., Ayappa K.G. (2021). Evaluating coarse-grained martini force-fields for capturing the ripple phase of lipid membranes. J Phys Chem B.

[40] Su J., Marrink S.J., Melo M.N. (2020). Localization preference of antimicrobial peptides on liquid-disordered membrane domains. Front Cell Dev Biol.

[41] Suarez-Leston F., Calvelo M., Tolufashe G.F., Muñoz A., Veleiro U., Porto C. (2022). SuPepMem: a database of innate immune system peptides and their cell membrane interactions. Comput Struct Biotechnol J.

[42] Parrinello M., Rahman A. (1998). Polymorphic transitions in single crystals: a new molecular dynamics method. J Appl Phys.

[43] Bussi G., Donadio D., Parrinello M. (2007). Canonical sampling through velocity rescaling. J Chem Phys.

[44] Hess B., Bekker H., Berendsen H.J.C., Fraaije J.G.E.M. (1997). LINCS: a linear constraint solver for molecular simulations. J Comput Chem.

[45] De Jong D.H., Baoukina S., Ingólfsson H.I., Marrink S.J. (2016). Martini straight: Boosting performance using a shorter cutoff and GPUs. Comput Phys Commun.

[46] Abraham M.J., Murtola T., Schulz R., Páll S., Smith J.C., Hess B. (2015). GROMACS: High performance molecular simulations through multi-level parallelism from laptops to supercomputers. Softw 1–2.

[47] PLUMED User’s Guide A portable plugin for free-energy calculations with molecular dynamics, (n.d.).

[48] Humphrey W., Dalke A., Schulten K. (1996). VMD: Visual molecular dynamics. J Mol Graph.

[49] Michaud-Agrawal N., Denning E.J., Woolf T.B., Beckstein O. (2011). MDAnalysis: A toolkit for the analysis of molecular dynamics simulations. J Comput Chem.

[50] Harris C.R., Millman K.J., van der Walt S.J., Gommers R., Virtanen P., Cournapeau D. (2020). Array programming with NumPy. Nature.

[51] Hunter J.D. (2007). Matplotlib: a 2D graphics environment. Comput Sci Eng.

[52] Esteban-Martín S., Salgado J. (2007). The dynamic orientation of membrane-bound peptides: bridging simulations and experiments. Biophys J.

[53] Fauchere J., Pliska V. (1983). Hydrophobic parameters II of amino acid side-chains from the partitioning of N-acetyl-amino acid amides. Eur J Med Chem.

[54] Garcia-Fandiño R., Piñeiro Á., Trick J.L., Sansom M.S.P. (2016). Lipid Bilayer Membrane Perturbation by Embedded Nanopores: A Simulation Study. ACS Nano.

[55] Lemons D.S., Ambegaokar V. (2003). An introduction to stochastic processes in physics. Am J Phys.

[56] Zhang L., Yethiraj A., Cui Q. (2014). Free energy calculations for the peripheral binding of proteins/peptides to an anionic membrane. 1. implicit membrane models. J Chem Theory Comput.

[57] Van Hilten N., Stroh K.S., Risselada H.J. (2022). Efficient quantification of lipid packing defect sensing by amphipathic peptides: comparing martini 2 and 3 with CHARMM36. J Chem Theory Comput.

[58] Granata D., Camilloni C., Vendruscolo M., Laio A. (2013). Characterization of the free-energy landscapes of proteins by NMR-guided metadynamics. Proc Natl Acad Sci USA.

[59] Hamelryck T., Borg M., Paluszewski M., Paulsen J., Frellsen J., Andreetta C. (2010). Potentials of mean force for protein structure prediction vindicated, formalized and generalized. PLoS One.

[60] Spiwok V., Kurečka M., Křenek A. (2022). Collective variable for metadynamics derived from alphafold output. Front Mol Biosci.

[61] Yin L.M., Edwards M.A., Li J., Yip C.M., Deber C.M. (2012). Roles of hydrophobicity and charge distribution of cationic antimicrobial peptides in peptide-membrane interactions. J Biol Chem.

[62] Wang C.K., Shih L.Y., Chang K.Y. (2017). Large-Scale Analysis of Antimicrobial Activities in Relation to Amphipathicity and Charge Reveals Novel Characterization of Antimicrobial Peptides. Molecules.

