# Supplementary material for: Unlocking the specificity of antimicrobial peptide interactions for membrane-targeted therapies
Source: Comput Struct Biotechnol J. 2024 Apr 12;25:61–74. doi: 10.1016/j.csbj.2024.04.022 (PMC11061258; doi:10.1016/j.csbj.2024.04.022)
Supplement: Supplementary file 1 — Supplementary material [file mmc1.docx]

**Supplementary information for**

**Unlocking the Specificity of Antimicrobial Peptide Interactions for Membrane-Targeted Therapies**

*Daniel Conde-Torres^1,2^, Martín Calvelo^3^, Carme Rovira^3,4^, Ángel Piñeiro^2,*^, Rebeca Garcia-Fandino^1,*^*

^1^Center for Research in Biological Chemistry and Molecular Materials, Departamento de Química Orgánica, Universidade de Santiago de Compostela, Campus Vida s/n, 15782 Santiago de Compostela, Spain.

^2^Departamento de Física Aplicada, Facultade de Física, Universidade de Santiago de Compostela, 15782 Santiago de Compostela, Spain

^3^Departament de Química Orgànica and Institut de Química Teòrica i Computacional (IQTCUB), Universitat de Barcelona, Barcelona, Spain.

^4^Institució Catalana de Recerca i Estudis Avançats (ICREA), Barcelona, Spain

^*^Corresponding Authors: [Angel.Pineiro@usc.es](mailto:Angel.Pineiro@usc.es), [rebeca.garcia.fandino@usc.es](mailto:rebeca.garcia.fandino@usc.es)

**Content:**

**1.- Thermodynamic description of the adsorption process**

**2.- Analysis of the Unbiased MD simulations**

**3.- Analysis of the Biased MD simulations**

**1.- Thermodynamic description of the adsorption process**

A detailed derivation of the thermodynamic partition coefficient for the distribution of helical peptides between a lipid membrane and the corresponding aqueous phase, taking as a reference the 1 M concentration standard state, is detailed in the main text.


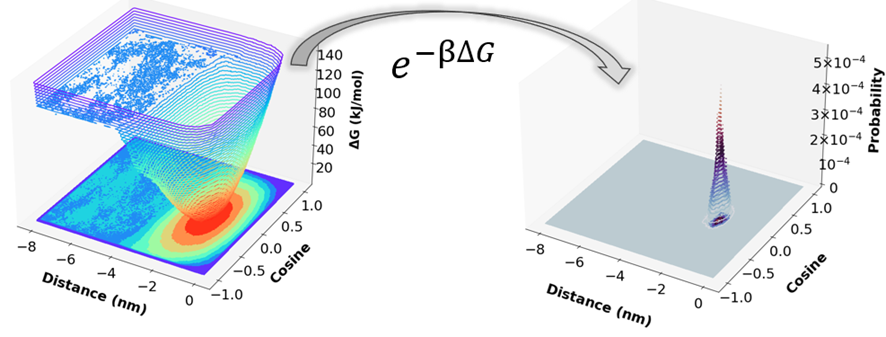


**Figure S1.-** Transformation from the energy profile using two CVs (the distance of the AMP to the membrane and the cosine of the tilt angle) into a probability distribution that, upon integration, leads to the partition coefficient.

**2.- Analysis of the Unbiased MD simulations**

**Table S1.-** Average distances in nm between the center of mass of the AMP and the center of the bilayer calculated over the last µs of all the free simulations. The composition of each membrane is indicated in Figure 2 of the main text.

| **Membrane** | **1Z64** | **2JMY** | **2K60** | **6C41** | **2MAG** | **2MAG-RC** | **2MAG-NC** | **2MAG-RM** |
| --- | --- | --- | --- | --- | --- | --- | --- | --- |
| **1** | 1.844±0.021 | 1.865±0.034 | 1.982±0.022 | 1.902±0.016 | 2.028±0.024 | 2.131±0.051 | 1.873±0.024 | 1.750±0.016 |
| **2** | 1.759±0.012 | 1.810±0.014 | 1.883±0.014 | 1.757±0.011 | 1.9003±0.0078 | 2.132±0.012 | 1.8572±0.0071 | 1.741±0.053 |
| **3** | 1.6672±0.0072 | 1.7930±0.0060 | 1.8773±0.0071 | 1.6897±0.0048 | 1.8483±0.0069 | 2.124±0.011 | 1.784±0.011 | 1.581±0.036 |

**Table S2.-** Average tilt angles in degrees, calculated as indicated in the main text, for all the AMPs calculated over the last µs of all the free simulations.

| **Membrane** |  | **1Z64** | **2JMY** | **2K60** | **6C41** | **2MAG** | **2MAG-RC** | **2MAG-NC** | **2MAG-RM** |
| --- | --- | --- | --- | --- | --- | --- | --- | --- | --- |
| **1** | **CANCER** | 191.2±4.3 | 188.5±1.9 | 172.82±0.63 | 183.21±0.92 | 185.4±1.1 | 214.5±2.6 | 178.0±1.2 | 184.9±4.3 |
| **2** | **BACTERIA** | 190.3±1.8 | 188.1±1.9 | 172.99±0.77 | 180.77±0.27 | 177.96±0.61 | 212.5±2.9 | 178.98±0.96 | 184.04±0.90 |
| **3** | **MAMMAL** | 184.7±1.4 | 188.8±2.4 | 172.80±0.73 | 183.7±1.2 | 178.63±0.75 | 226.6±1.5 | 177.85±0.75 | 180.3±2.0 |

**Table S3.-** Average spin angles in degrees, calculated as indicated in the main text, for all the AMPs calculated over the last µs of all the free simulations.

| **Membrane** |  | **1Z64** | **2JMY** | **2K60** | **6C41** | **2MAG** | **2MAG-RC** | **2MAG-NC** | **2MAG-RM** |
| --- | --- | --- | --- | --- | --- | --- | --- | --- | --- |
| **1** | **CANCER** | 88.36±0.84 | 91.7±1.0 | 86.87±0.52 | 86.52±0.63 | 86.57±0.63 | 82.9±1.6 | 86.3±1.1 | 85.5±1.3 |
| **2** | **BACTERIA** | 90.81±0.34 | 94.02±0.41 | 85.66±0.12 | 87.74±0.26 | 85.28±0.13 | 74.5±1.3 | 85.30±0.31 | 86.9±1.8 |
| **3** | **MAMMAL** | 91.44±0.24 | 93.20±0.26 | 86.24±0.18 | 86.28±0.23 | 86.13±0.39 | 70.6±1.5 | 86.28±0.15 | 85.8±1.4 |


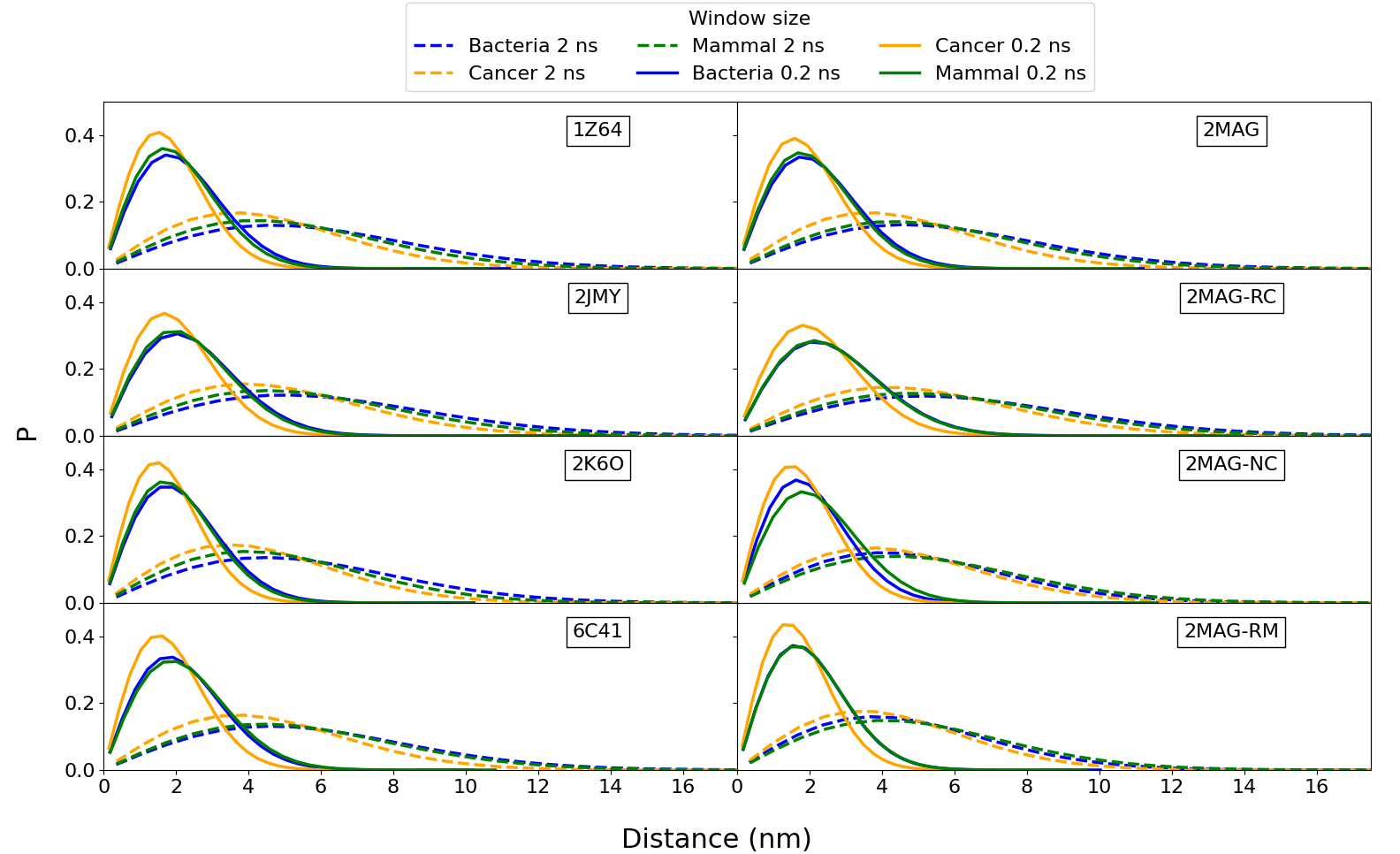


**Figure S2.-** Lateral displacement probability distributions for the peptide corresponding to time windows of 0.2 ns (solid lines) and 2 ns (dashed lines) for the membrane model 1 (blue), 2 (orange) and 3 (green) membrane models. The values of the diffusion coefficients obtained from these distributions and also for those using a time window of 100 ns are in table S4.

**Table S4.-** Diffusion coefficients in 10^−7^ cm^2^/s calculated, as indicated in the methods section, from the lateral displacement probability distributions of Fig. S2. The composition of each membrane is indicated in Figure 2 of the main text.

| **Membrane** | **Time** | **1Z64** | **2JMY** | **2K60** | **6C41** | **2MAG** | **2MAG-RC** | **2MAG-NC** | **2MAG-RM** |
| --- | --- | --- | --- | --- | --- | --- | --- | --- | --- |
| **1** | 100 | 0.777±0.044 | 0.806±0.036 | 0.636±0.068 | 0.756±0.022 | 0.573±0.016 | 0.742±0.023 | 0.743±0.031 | 0.758±0.018 |
|  | 2 | 3.286±0.061 | 3.83±0.18 | 3.038±0.060 | 3.41±0.31 | 3.28±0.15 | 4.36±0.13 | 3.39±0.14 | 2.95±0.12 |
|  | 0.2 | 5.52±0.12 | 6.85±0.44 | 5.20±0.15 | 5.66±0.54 | 6.04±0.35 | 8.42±0.40 | 5.46±0.29 | 4.79±0.26 |
| **2** | 100 | 0.939±0.040 | 0.75±0.11 | 0.860±0.026 | 0.830±0.024 | 0.740±0.029 | 0.866±0.055 | 0.741±0.023 | 0.5953±0.0068 |
|  | 2 | 5.443±0.041 | 6.19±0.29 | 4.984±0.097 | 5.35±0.53 | 5.32±0.25 | 6.513±0.094 | 4.07±0.24 | 3.61±0.25 |
|  | 0.2 | 7.924±0.074 | 9.87±0.60 | 7.52±0.14 | 8.01±0.62 | 8.21±0.36 | 11.60±0.25 | 6.81±0.55 | 6.60±0.37 |
| **3** | 100 | 0.736±0.020 | 0.666±0.023 | 0.714±0.055 | 0.787±0.041 | 0.722±0.082 | 0.702±0.048 | 0.879±0.055 | 0.653±0.022 |
|  | 2 | 4.41±0.11 | 5.03±0.36 | 3.88±0.10 | 4.87±0.43 | 4.63±0.31 | 5.68±0.20 | 4.71±0.33 | 4.16±0.25 |
|  | 0.2 | 7.09±0.15 | 9.26±0.73 | 6.97±0.10 | 8.61±0.59 | 7.63±0.37 | 11.34±0.67 | 8.31±0.46 | 6.65±0.37 |

**
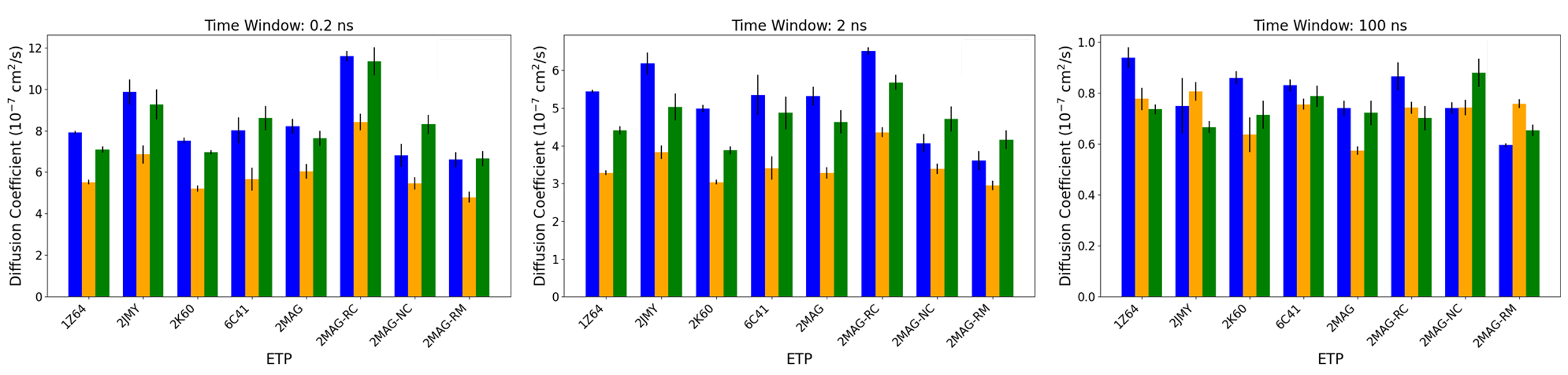
**

**Figure S3.-** Diffusion coefficients calculated from the lateral displacement probability distributions of Fig. S2, taken from Table S4. Membrane models 1, 2 and 3 are in orange, blue and green color. The composition of each membrane is indicated in Figure 2 of the main text.

**3.- Analysis of the Biased MD simulations**

**
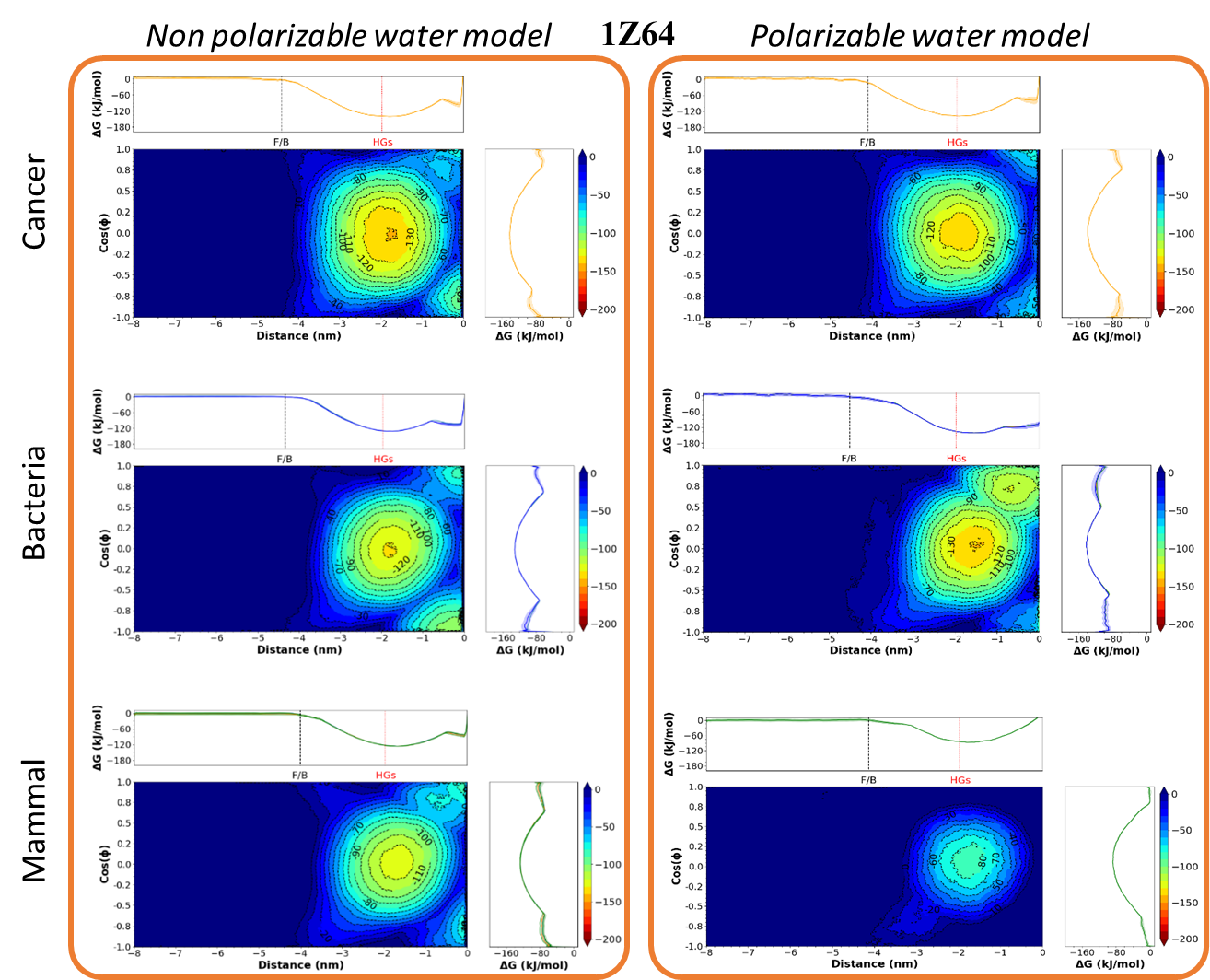
**

**Figure S4.-** 2D-PMF profiles for the system **1Z64** obtained from Metadynamics simulations using the distance between the center of mass of the peptide and the center of the bilayer (z) and the cosine of the tilt angle as collective variables. The results obtained for the membrane models 1, 2 and 3 are in the top, central and bottom plots, respectively. The PMFs for the simulations with non-polarizable and polarizable water models are shown, as indicated in the labels. The colormap represents the value of the Gibbs energy. The projection of the PMF on each CV (see methods section) are represented on the top and right of each colormap. The dotted red line in the projection on the distance shows the position of the membrane phosphorous while the dashed black line represents the distance beyond which the interaction between the AMP and the membrane are negligible (F/B correspond to “free” and “bound” regions on the left and right, respectively).

*
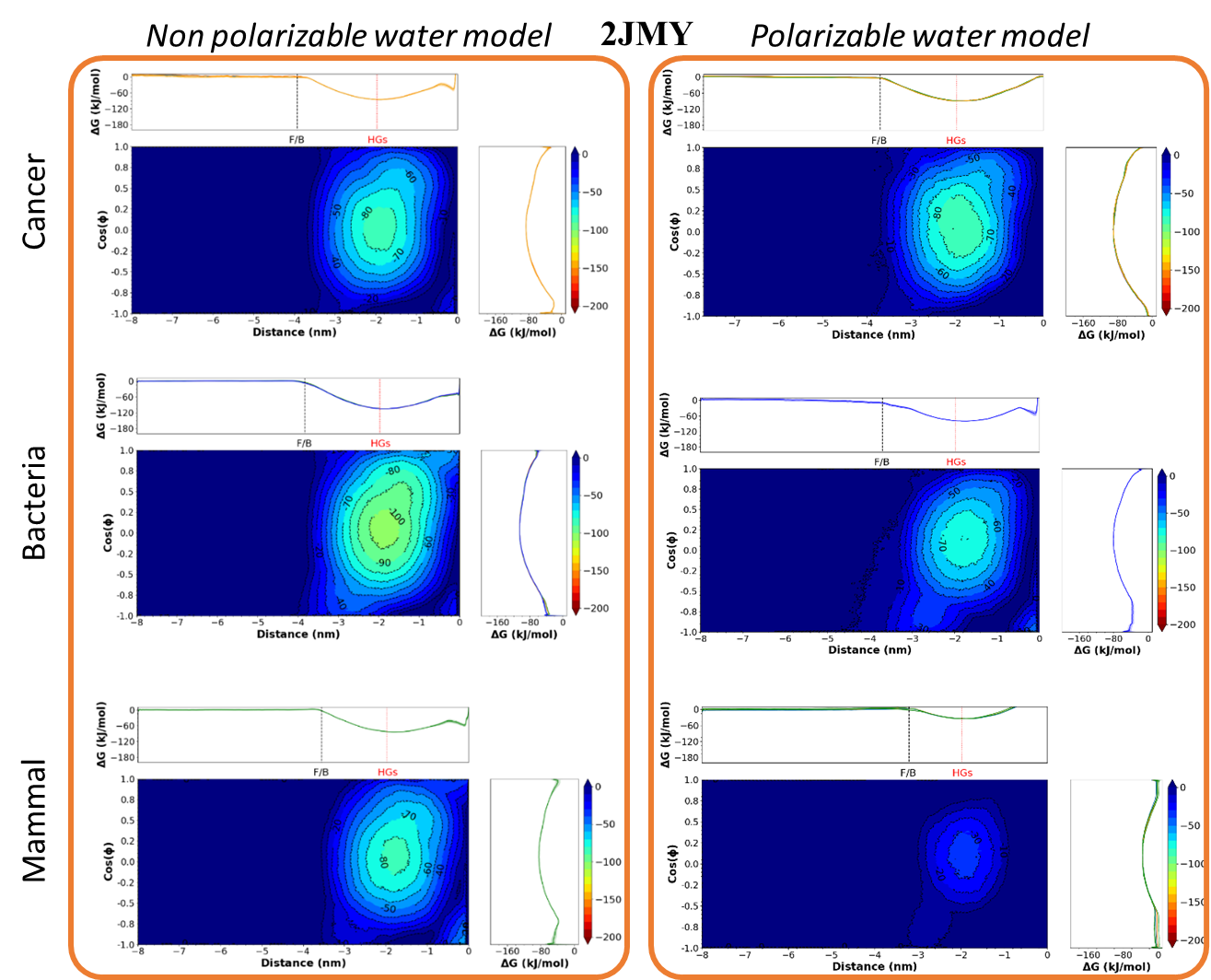
*

**Figure S5.-** 2D-PMF profiles for the system **2JMY** obtained from Metadynamics simulations using the distance between the center of mass of the peptide and the center of the bilayer (z) and the cosine of the tilt angle as collective variables. The results obtained for the membrane models 1, 2 and 3 are in the top, central and bottom plots, respectively. The PMFs for the simulations with non-polarizable and polarizable water models are shown, as indicated in the labels. The colormap represents the value of the Gibbs energy. The projection of the PMF on each CV (see methods section) are represented on the top and right of each colormap. The dotted red line in the projection on the distance shows the position of the membrane phosphorous while the dashed black line represents the distance beyond which the interaction between the AMP and the membrane are negligible (F/B correspond to “free” and “bound” regions on the left and right, respectively).

*
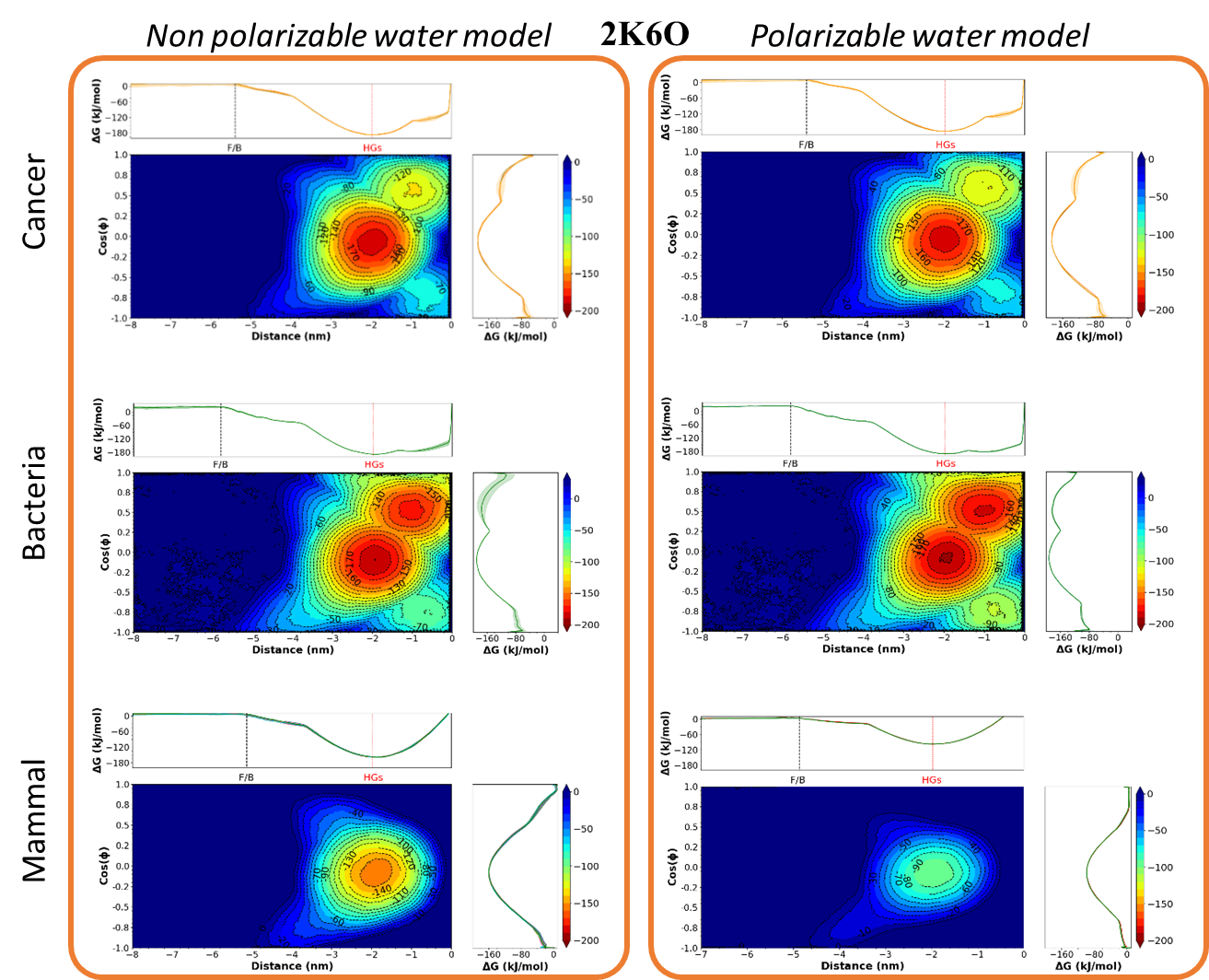
*

**Figure S6.-** 2D-PMF profiles for the system **2K6O** obtained from Metadynamics simulations using the distance between the center of mass of the peptide and the center of the bilayer (z) and the cosine of the tilt angle as collective variables. The results obtained for the membrane models 1, 2 and 3 are in the top, central and bottom plots, respectively. The PMFs for the simulations with non-polarizable and polarizable water models are shown, as indicated in the labels. The colormap represents the value of the Gibbs energy. The projection of the PMF on each CV (see methods section) are represented on the top and right of each colormap. The dotted red line in the projection on the distance shows the position of the membrane phosphorous while the dashed black line represents the distance beyond which the interaction between the AMP and the membrane are negligible (F/B correspond to “free” and “bound” regions on the left and right, respectively).

*
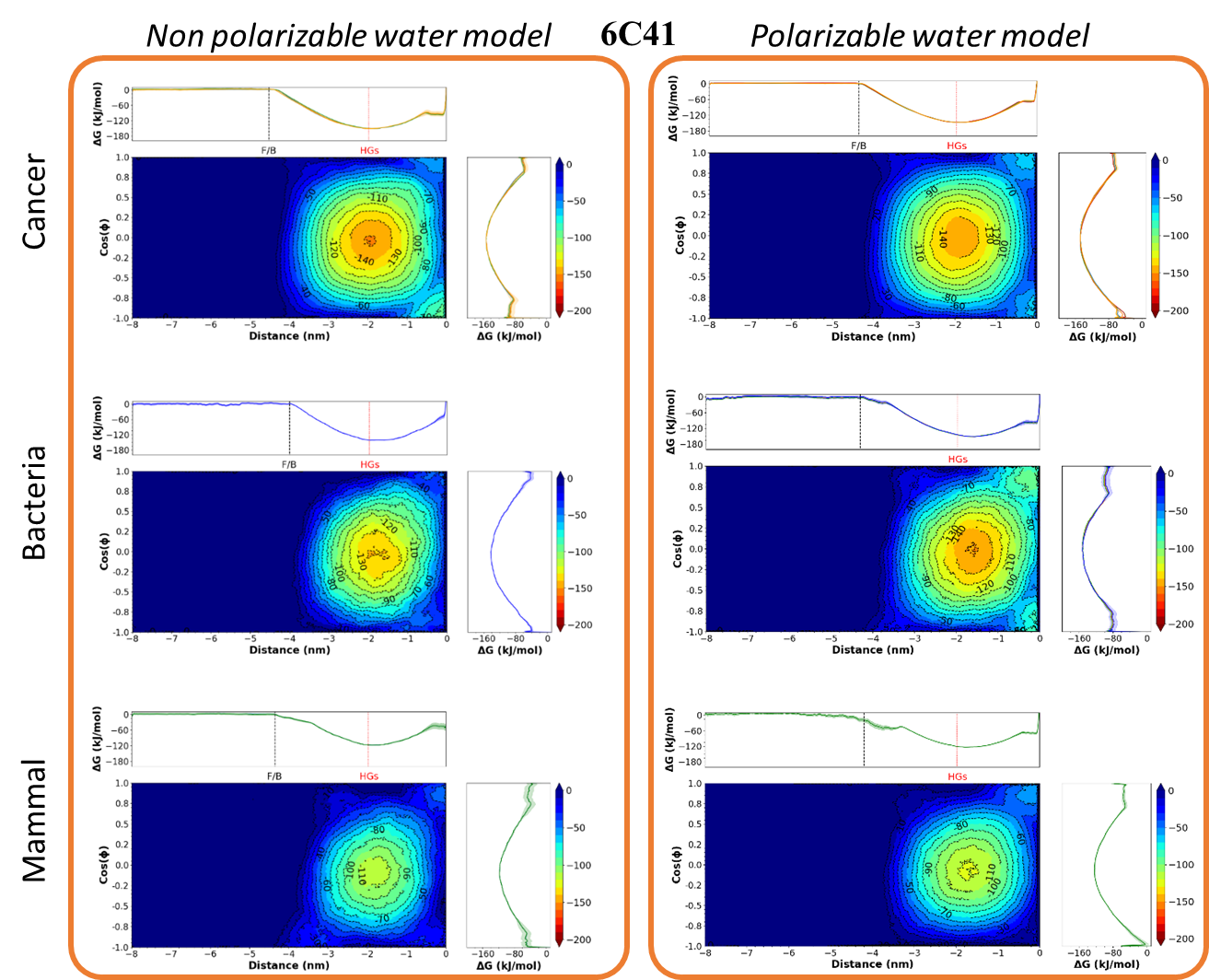
*

**Figure S7.-** 2D-PMF profiles for the system **6C41** obtained from Metadynamics simulations using the distance between the center of mass of the peptide and the center of the bilayer (z) and the cosine of the tilt angle as collective variables. The results obtained for the membrane models 1, 2 and 3 are in the top, central and bottom plots, respectively. The PMFs for the simulations with non-polarizable and polarizable water models are shown, as indicated in the labels. The colormap represents the value of the Gibbs energy. The projection of the PMF on each CV (see methods section) are represented on the top and right of each colormap. The dotted red line in the projection on the distance shows the position of the membrane phosphorous while the dashed black line represents the distance beyond which the interaction between the AMP and the membrane are negligible (F/B correspond to “free” and “bound” regions on the left and right, respectively).

*
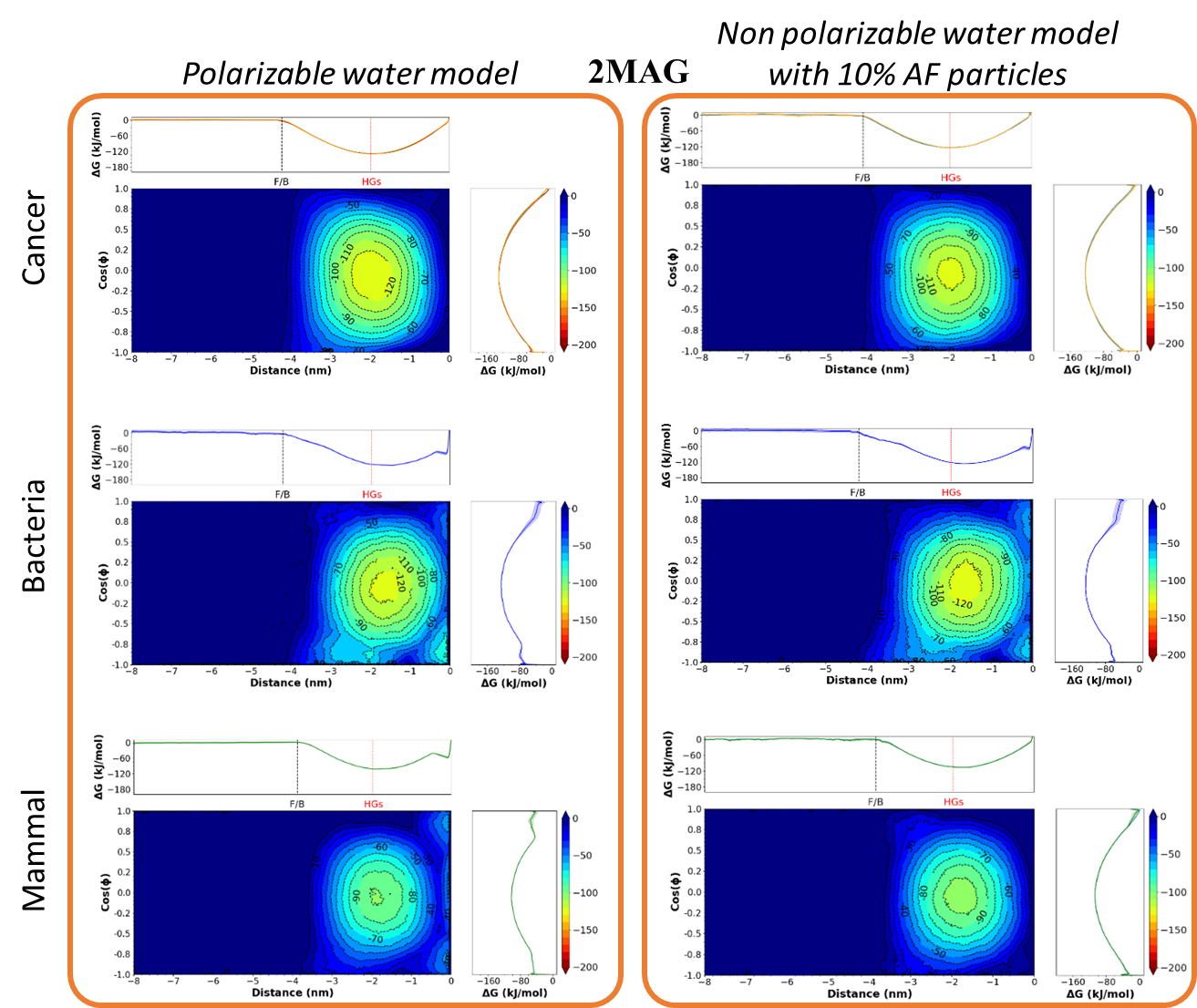
*

**Figure S8.-** 2D-PMF profiles for the system **2MAG** obtained from Metadynamics simulations using the distance between the center of mass of the peptide and the center of the bilayer (z) and the cosine of the tilt angle as collective variables. The results obtained for the membrane models 1, 2 and 3 are in the top, central and bottom plots, respectively. The PMFs for the simulations with pure non-polarizable water and also adding 10% of antifreeze particles are shown, as indicated in the labels. The colormap represents the value of the Gibbs energy. The projection of the PMF on each CV (see methods section) are represented on the top and right of each colormap. The dotted red line in the projection on the distance shows the position of the membrane phosphorous while the dashed black line represents the distance beyond which the interaction between the AMP and the membrane are negligible (F/B correspond to “free” and “bound” regions on the left and right, respectively).

*
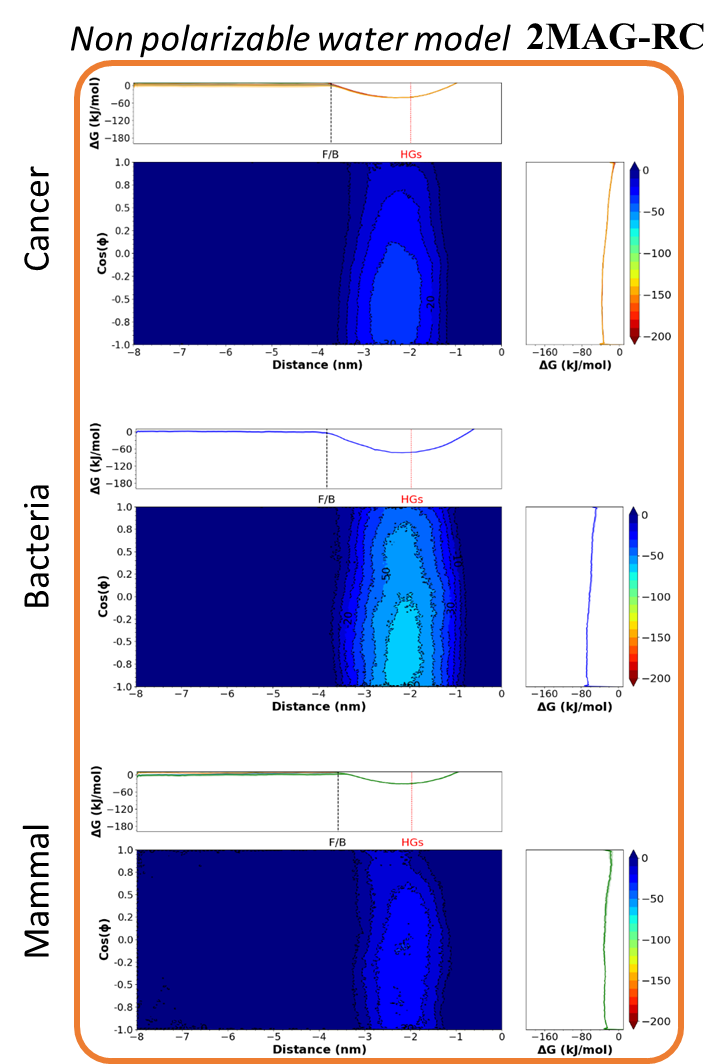
*

**Figure S9.-** 2D-PMF profiles for the system **2MAG-RC** obtained from Metadynamics simulations using the distance between the center of mass of the peptide and the center of the bilayer (z) and the cosine of the tilt angle as collective variables. The results obtained for the membrane models 1, 2 and 3 are in the top, central and bottom plots, respectively. The PMFs for the simulations with pure non-polarizable water are shown, as indicated in the label. The colormap represents the value of the Gibbs energy. The projection of the PMF on each CV (see methods section) are represented on the top and right of each colormap. The dotted red line in the projection on the distance shows the position of the membrane phosphorous while the dashed black line represents the distance beyond which the interaction between the AMP and the membrane are negligible (F/B correspond to “free” and “bound” regions on the left and right, respectively).

*
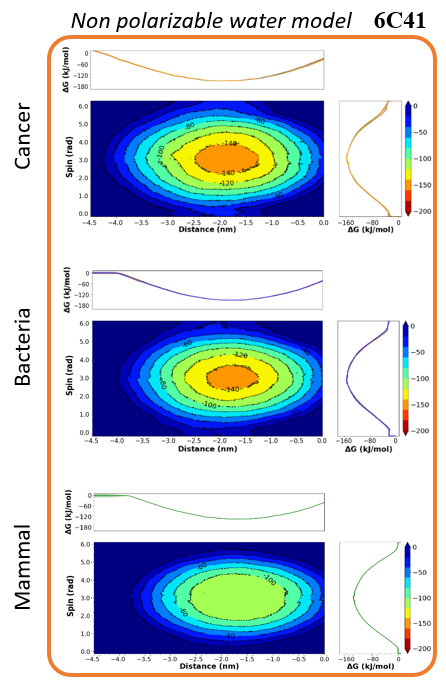
*

**Figure S10.-** 2D-PMF profiles for the system **6C41** obtained from Metadynamics simulations using the distance between the center of mass of the peptide and the center of the bilayer (z) and the spin angle as collective variables. The results obtained for the membrane models 1, 2 and 3 are in the top, central and bottom plots, respectively. The PMFs for the simulations with pure non-polarizable water are shown, as indicated in the label. The colormap represents the value of the Gibbs energy. The projection of the PMF on each CV (see methods section) are represented on the top and right of each colormap. The dotted red line in the projection on the distance shows the position of the membrane phosphorous while the dashed black line represents the distance beyond which the interaction between the AMP and the membrane are negligible (F/B correspond to “free” and “bound” regions on the left and right, respectively).

**Table S5.-** Standard Gibbs energies obtained from the Metadynamics simulations using the cosine of the tilt angle and the distance between the center of mass of the peptide and the center of the membrane as CVs.

|  |  |  | Non Polarizable | Polarizable |
| --- | --- | --- | --- | --- |
|  | Membrane |  | water model | water model |
| AMP | Model |  | ΔG^0^ kJ/mol | ΔG^0^ kJ/mol |
|  | 1 |  | −121.5 ± 3.1 | −119.2 ± 3 |
| **1Z64** | 2 |  | −117.5 ± 3.2 | −120.1 ± 2.3 |
|  | 3 |  | −113.5 ± 2.6 | −72.4 ± 1.9 |
|  | 1 |  | −73.2 ± 1.6 | −76.6 ± 2.4 |
| **2JMY** | 2 |  | −91.9 ± 2.1 | −86.8 ± 2.1 |
|  | 3 |  | −71.2 ± 1.8 | −22.1 ± 1.3 |
|  | 1 |  | −171.9 ± 3.3 | −172.1 ± 3.6 |
| **2K6O** | 2 |  | −175.3 ± 4.2 | −174.6 ± 3.4 |
|  | 3 |  | −142.3 ± 3.2 | −84.5 ± 2.8 |
|  | 1 |  | −137 ± 2.5 | −135.1 ± 2.4 |
| **6C41** | 2 |  | −134.2 ± 2.7 | −136.4 ± 2.5 |
|  | 3 |  | −108.2 ± 2.3 | −104.6 ± 2.1 |
|  | 1 |  | −115.7 ± 3.1 | −116.5 ± 2.8 |
| **2MAG** | 2 |  | −110.6 ± 3.2 | −113.1 ± 3.1 |
|  | 3 |  | −89.1 ± 2.6 | −85.3 ± 2.3 |
|  | 1 |  | −20.7 ± 1.1 |  |
| **2MAG-RC** | 2 |  | −55.4 ± 1.3 |  |
|  | 3 |  | −19.4 ± 1.8 |  |
|  | 1 |  | −112.9 ± 2.1 |  |
| **2MAG-NC** | 2 |  | −111.3 ± 2.4 |  |
|  | 3 |  | −88.5 ± 3.0 |  |
|  | 1 |  | −104.4 ± 4.1 |  |
| **2MAG-RM** | 2 |  | −106.1 ± 5.2 |  |
|  | 3 |  | −79.8 ± 4.6 |  |
|  | 1 |  | −110.5 ± 2.5 |  |
| **2MAG-AF** | 2 |  | −112.1 ± 3.1 |  |
|  | 3 |  | −90.5 ± 2.4 |  |
